# Targeting the RAS/RAF/MAPK pathway for cancer therapy: from mechanism to clinical studies

**DOI:** 10.1038/s41392-023-01705-z

**Published:** 2023-12-18

**Authors:** Md Entaz Bahar, Hyun Joon Kim, Deok Ryong Kim

**Affiliations:** 1 Department of Biochemistry and Convergence Medical Sciences and Institute of Medical Science, Gyeongsang National University, College of Medicine, Jinju, South Korea; 2 Department of Anatomy and Convergence Medical Sciences and Institute of Medical Science, Gyeongsang National University, College of Medicine, Jinju, South Korea

**Keywords:** Drug development, Metastasis

## Abstract

Metastatic dissemination of solid tumors, a leading cause of cancer-related mortality, underscores the urgent need for enhanced insights into the molecular and cellular mechanisms underlying metastasis, chemoresistance, and the mechanistic backgrounds of individuals whose cancers are prone to migration. The most prevalent signaling cascade governed by multi-kinase inhibitors is the mitogen-activated protein kinase (MAPK) pathway, encompassing the RAS–RAF–MAPK kinase (MEK)–extracellular signal-related kinase (ERK) pathway. RAF kinase is a primary mediator of the MAPK pathway, responsible for the sequential activation of downstream targets, such as MEK and the transcription factor ERK, which control numerous cellular and physiological processes, including organism development, cell cycle control, cell proliferation and differentiation, cell survival, and death. Defects in this signaling cascade are associated with diseases such as cancer. RAF inhibitors (RAFi) combined with MEK blockers represent an FDA-approved therapeutic strategy for numerous *RAF*-mutant cancers, including melanoma, non-small cell lung carcinoma, and thyroid cancer. However, the development of therapy resistance by cancer cells remains an important barrier. Autophagy, an intracellular lysosome-dependent catabolic recycling process, plays a critical role in the development of RAFi resistance in cancer. Thus, targeting RAF and autophagy could be novel treatment strategies for *RAF*-mutant cancers. In this review, we delve deeper into the mechanistic insights surrounding RAF kinase signaling in tumorigenesis and RAFi-resistance. Furthermore, we explore and discuss the ongoing development of next-generation RAF inhibitors with enhanced therapeutic profiles. Additionally, this review sheds light on the functional interplay between RAF-targeted therapies and autophagy in cancer.

## Introduction

The mitogen-activated protein kinase (MAPK) pathway transmits extracellular signals from the membrane to intracellular destinations and is involved in various biological functions.^1^ The MAPK pathway is dysregulated in many RAS-associated cancers. RAS mutations result in the constitutive activation of the MAPK pathway, leading to uncontrolled cell proliferation and resistance to apoptosis-inducing drugs.^2,3^ Although many RAS inhibitors have been isolated and studied, the development of drugs targeting RAS is limited by a lack of well-defined druggable nooks and cavities on the RAS surface.^4^ However, interrupting signals between RAS and downstream effectors, such as the RAF–MAPK kinase (MEK)–extracellular signal-related kinase (ERK) pathway, could represent a new therapeutic strategy for *RAS*-driven cancers.^5–7^

The RAF protein family consists of three serine (Ser)/threonine (Thr) kinases (ARAF, BRAF, and CRAF) that act as mediators between membrane-bound RAS-GTPases and downstream kinases, such as MEK and ERK, in the MAPK signaling pathway.^8^ RAF proteins coordinate various cellular responses by regulating cytoplasmic and nuclear activities, such as cell cycle progression, proliferation, metabolism, migration, differentiation, and apoptosis.^9,10^ RAF is highly conserved in mammals, and *RAF* mutations are associated with many human cancers, including melanoma, breast cancer, ovarian cancer, colon cancer, thyroid cancer, and prostate cancer.^11^ Mutations in BRAF and RAS that dysregulate MAPK signaling are strongly associated with human malignancies.^12^ All members of the RAF family interact with RAS; however, this contact alone is insufficient to activate RAF. For example, several *RAS* mutants, such as *RASV12Y32F* and *RASV12T35S* are insufficient to activate RAF in vitro, suggesting that RAF kinase activation requires other factors.^13^ A recent study indicates that the activation of RAF necessitates dimerization, and exploring RAF activation is currently being viewed as a potential target for therapeutic intervention in several clinical contexts, including diverse cancer types.^10^

Numerous RAF inhibitors are considered potential therapeutic agents, eliciting high levels of responses in various *RAF*-mutant carcinomas.^14,15^ However, single-agent therapies targeting RAF have not resulted in significant long-term survival benefits due to the frequent development of drug resistance, often associated with mutational changes in MAPK components that result in the reactivation of the MAPK pathway.^16,17^ Combination therapeutic strategies using both RAF and MEK inhibitors may represent a more effective treatment strategy in patients with advanced or metastatic *RAF*-mutant carcinomas.^18–21^ Although this approach has demonstrated potential efficacy in preclinical studies, clinical testing has not demonstrated durable responses, and a single-arm study demonstrated that this strategy is associated with a predictable pattern of adverse effects due to the substantial inhibition of multiple paralogs.^22,23^ Identifying key downstream signals in the MAPK pathway is essential for minimizing paralog redundancy and cascade interactions, which may limit both the cancerous activity of RAF and drug toxicity in normal cells.

Autophagy, an intracellular catabolic process, may assist cancer cells in evading from RAFi, as many RAFi-resistant cells exhibit enhanced autophagic activity.^24–26^ Both preclinical and clinical data suggest that inhibiting both autophagy and MAPK pathway activity may serve as a novel and effective treatment strategy for *BRAF* and *KRAS*-mutant cancers.^24,25,27^ In particular, Chih-Shia Lee’s group showed that targeting both *RAF* and autophagy genes results in the best therapeutic outcomes. The inhibition of BRAF or CRAF, together with ATG7 inhibition, was found to be a viable treatment strategy for RAS-driven tumors.^27^ Understanding the mechanisms underlying RAFi-induced autophagy in the setting of recurrent somatic genetic alterations and RAF mutations could offer a “precision medicine” paradigm for diagnosing and treating tumors, including *RAF*-mutant tumors. This review focuses on potentially unique therapeutic approaches that target the basic components of RAF signaling and autophagy in RAS-dependent and RAS-independent cancers.

## The discovery and major developments of RAS/RAF/MAPK in health and disease

The RAF/RAF/MEK/ERK signaling cascade is a well-established MAPK pathway in cell biology that governs several crucial cellular processes such as development, differentiation, proliferation, and death.^28^ With this cascade, various isoforms of RAS, RAF, MEK, and ERK exhibit differences in efficacy, function, and, notably, carcinogenic potential.

**Fig. 1:**
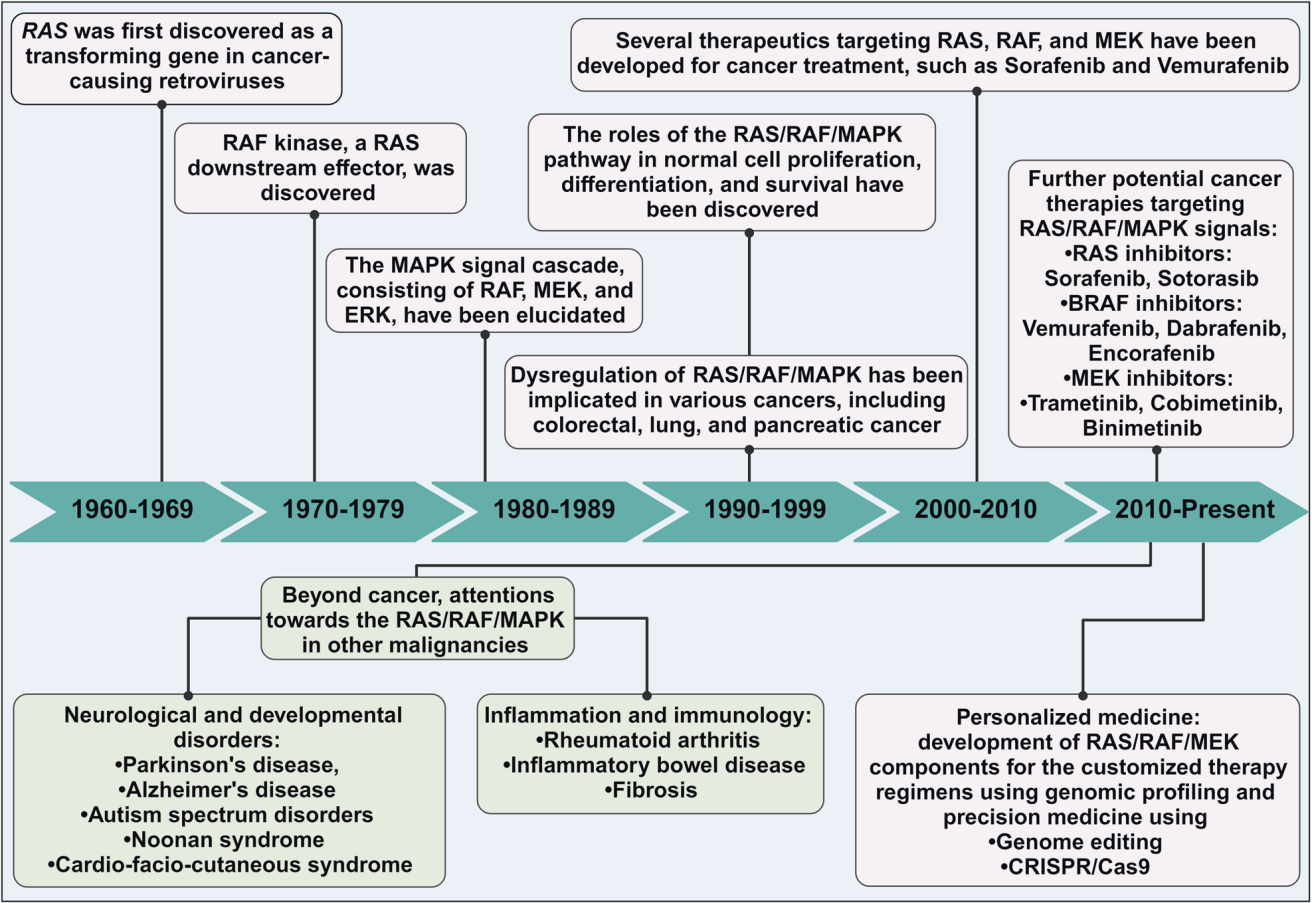
Historical events of the discovery and development of the RAS/RAF/MAPK pathway in health and diseases. The journey of the MAPK signal cascade commenced in 1960s with the groundbreaking discovery of the viral *RAS* gene. Subsequently, in 1992, the identification of RAF as both an upstream kinase activator of MEK and a RAS effector marked significant milestones. These pivotal findings culminated in the comprehensive definition of the entire MAPK signaling pathway. Over time, the MAPK signal emerged as a critical components in the development of therapeutic strategies for combating cancer. Each of these major milestones in the RAS/RAF/MAPK discovery is represented within its respective box. This figure was created with BioRender.com

Our understanding of oncogenic potential began with the discovery of the highly carcinogenic Harvey murine sarcoma virus^29^ in 1964 and the Kirsten murine sarcoma virus^30^ in 1967. In the late 1970s and early 1980s, groundbreaking studies by Scolnick and colleagues identified the cellular origins of viral *H-RAS* and *K-RAS* genes^31^, and an avian homolog MH2 retrovirus^32^ in 1984, and an avian homolog MH2 retrovirus^32^ in 1984, respectively. Later, these two oncogenes are known as the first rapidly accelerated fibrosarcoma (*RAF*) gene with serine/threonine kinase activity.^33^ Subsequently, *Raf-1* gene product (named as CRAF), a cellular counterpart of *v-Raf*, and other cellular counterparts such as *C-Raf-1* and *C-Raf-2* genes were cloned and sequenced in 1985.^34^ ARAF and BRAF, two additional members of the RAF family, were reported in 1986 and 1988, respectively.^35^ In 1988, MAPK, initially named microtubule-associated protein-2 protein kinase (MAP-2 kinase), was identified in mammalian cells^36^, and subsequently in yeast cells.^37^ MEK (mitogen-activated protein kinase kinase) and ERK (mitogen-activated protein kinase), both cytoplasmic protein kinases activated by mitogens, were discovered in the 1990s^38^. Further, RAF protein was functionally identified as a direct MEK activator^39^ in 1992 and a RAS effector in 1993.^40^ These findings marked the beginning of the MAPK cascade era, where it become evident that MAPK kinase signaling cascades play a pivotal role in initiating proliferative and oncogenic activities.^41^ In 2005, the U.S. Food and Drug Administration (FDA) approved Nexavar (Sorafenib), an oral multi-kinase inhibitor targeting the MAPK pathway, for the treatment of hepatocellular carcinoma (HCC), renal cell carcinoma (RCC), and thyroid carcinoma (TC).^42^ Subsequently, Sorafenib was proposed as a MAPK pathway inhibitor for malignant peripheral nerve sheath tumors (MPNSTs).^43^ Vemurafenib (Zelboraf), a potent *BRAF*^*V600E*^ mutant inhibitor, was synthesized in early 2005 and received FDA approval for the treatment of metastatic and late-stage melanoma in 2011.^44^ Following this, FDA approval were granted for two BRAF inhibitors, Dabrafenib (Tafinlar) in 2013 and Encorafenib (Braftovi) in 2018. Trametinib (Mekinist) was approved in 2013 as a single-agent oral treatment for unresectable or metastatic melanoma in adult patients with *BRAF*^*V600E*^ or *BRAF*^*V600K*^ mutations.^45^ From 2014 to 2023, Trametinib, in combination with Dabrafenib, received FDA approval for the treatment of various solid tumors, including melanoma, non-small cell lung cancer (NSCLC), anaplastic thyroid cancer (ATC), and, low-grade glioma (LGG).^46^ In addition, two MEK inhibitors, Cotellic (Cobimetinib) and Mektovi (Binimetinib) were approved in 2015 and 2018, respectively, for the treatment of melanoma, either as a single-agent or in combination with other MAPK inhibitors.^47^ Ongoing preclinical and clinical investigations underscore the potential of the RAS/RAF/MAPK pathway as a significant therapeutic target, particularly in the era of precision medicine, with a focus on combination treatments.^48^ For example, a CRISPR/cas9 gene deletion study in lung cancer cells revealed that the deletion of KEAP1, in the presence of specific RAS/RAF/MAPK pathway inhibitors, alters cell metabolism and enables cells to proliferate without MAPK signaling.^49^ These major milestones are depicted in Fig. 1.

Beyond cancer, ongoing studies aim to develop new treatments targeting RAS/RAF/MAPK for various disorders, including neurological, developmental, and metabolic diseases. Neurological disorders such as autism spectrum disorder (ASD), Parkinson’s disease (PD), Alzheimer’s disease (AD), Cardio-Facio-cutaneous (CFC), and Noonan syndromes (NS) have been linked to abnormalities in the regulation of the MAPK signaling pathway.^50^ Moreover, the RAS/RAF/MAPK pathway is gaining attention as a potential target for the development of novel anti-inflammatory drugs, with implications for conditions like rheumatic arthritis (RA)^51^, inflammatory bowel disease (IBD)^52^ and pulmonary fibrosis (PF).^53^

## MAPK signaling

**Fig. 2:**
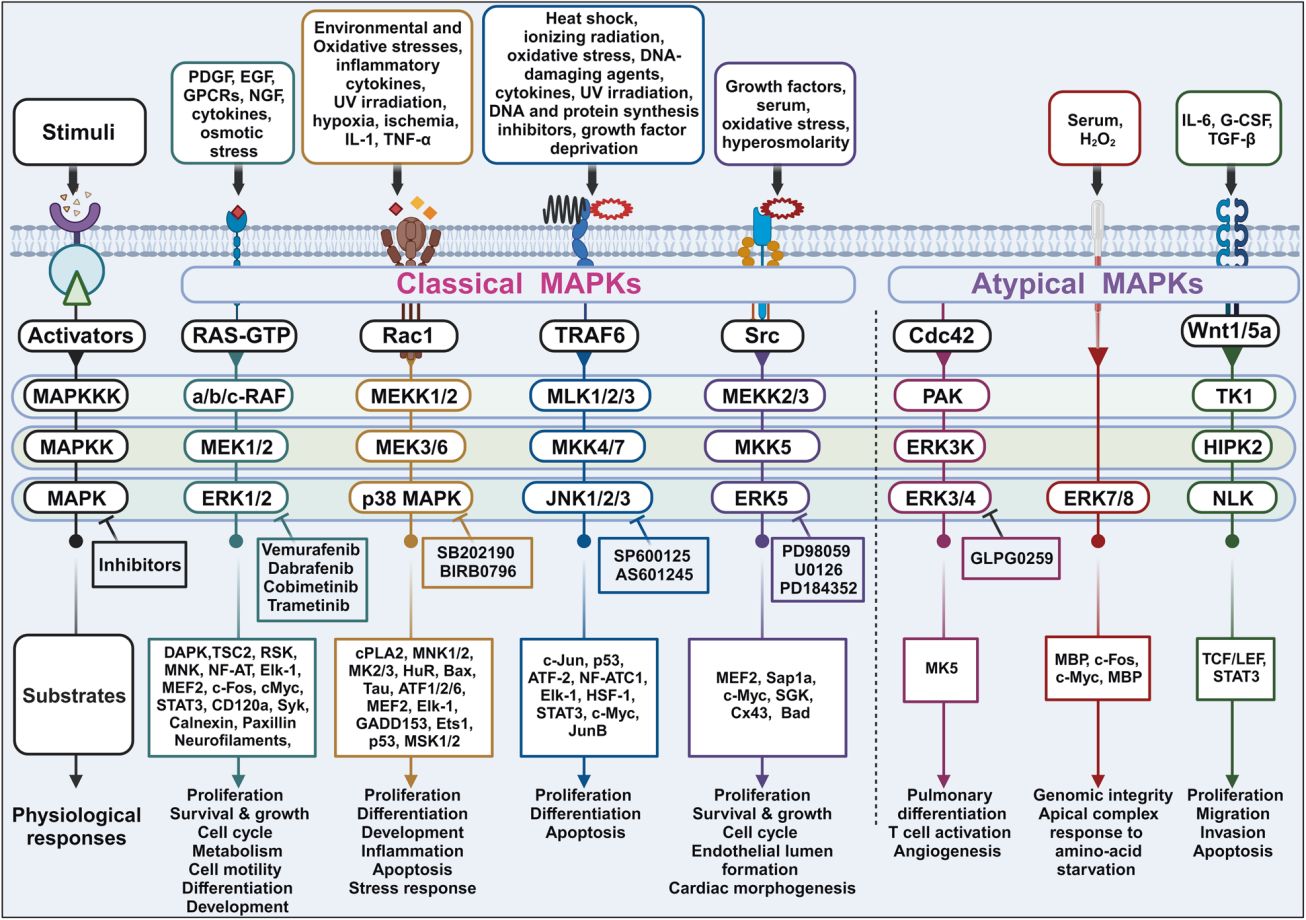
Mitogen-activated protein kinase (MAPK) cascades and their physiological functions. All cascades consist of three-layered core-signaling pathways in which each kinase is consecutively activated, and MAPK components are highly conserved. The first layer consists of MAPK kinase kinases (MAPKKKs or MEKKs), which are activated by stimuli and phosphorylate and activate MAPK kinases (MAPKKs or MEKs). MAPKKs are dual-specificity kinases that can phosphorylate threonine or tyrosine residues to activate the terminal serine/threonine MAPK, leading to the activation of multiple cytoplasmic and nuclear proteins involved in various biological functions. This figure was created with BioRender.com

MAPKs are Ser/Thr kinases that play various roles in cellular responses to stimuli, including mitogens, osmotic stress, heat shock, and proinflammatory cytokines. MAPKs are involved in many cellular processes, such as proliferation, gene expression, differentiation, mitosis, cell survival, and apoptosis.^54^ Mammals possess four primary MAPKs: (1) ERK1/2, (2) c-Jun N-terminal kinase (JNK)1–3, (3) p38, and (4) ERK5.^55–57^ In addition to these four primary MAPKs, numerous atypical MAPKs (e.g., ERK 3/4, ERK 7/8, and Nemo-like kinase [NLK]) have been identified with less well-defined roles and unique mechanisms of activation.^58–60^ MAPK cascades consist of a signaling relay that is partially regulated by phosphorylation and typically involves three consecutive protein kinases: MEK kinase (MAPKKK), MEK, and MAPK (Fig. 2). MAPK cascades are activated by cell-surface receptors via cytoplasmic signaling proteins, and these signaling pathways are often dysregulated in human cancers. ERK1 and ERK2 are frequently investigated by researchers worldwide due to their critical roles in cell proliferation and survival. The JNK and p38 MAPK pathways primarily play roles in responding to cellular stress and regulating apoptosis. In contrast, the most extensively studied RAS/RAF/MAPK pathway holds a central position in governing cell proliferation and differentiation, serving as a vital component of the cellular signal transduction network. Consequently, proteins involved in the RAS/RAF/MAPK cascade have frequently been targeted in cancer drug discovery, leading to the clinical development of protein kinase inhibitors.^61^

### RAS/RAF/MAPK signaling: structure, upstream activator and downstream effectors

The ERK (MAPK) kinase plays a pivotal role within the RAS/RAF/MAPK signal transduction pathway, exerting control over various facets of cellular metabolism in cancer cells.^62,63^ Consequently, when it comes to development of anticancer drugs, the focus largely centers on three key upstream regulators and ERK protein in the ERK pathway: RAS (upstream activator of RAF), RAF (direct effector of RAS and activator of MEK), MEK (functioning as MAPKK), and ERK (as MAPK).

**Fig. 3:**
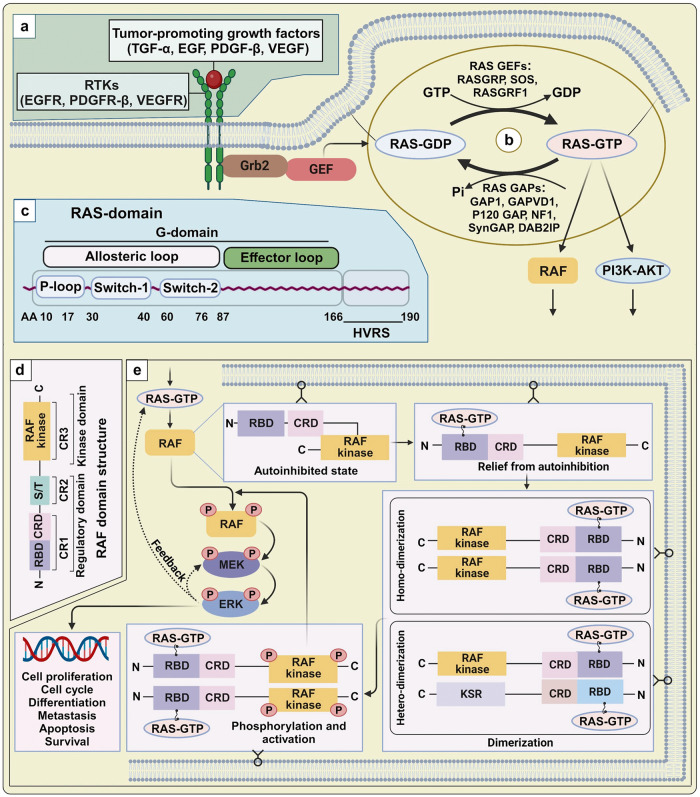
Structure and activation mechanism of RAS and RAF kinase in the RAS/RAF/MAPK signal cascade. **a** RAS upstream components. Various mitogens including TGF-α, EGF, VEGF, and PDGF-β bind to their own receptors and lead to RAS activation and subsequent stimulation of the MAPK pathway. **b** GTPase cycle. GEFs stimulates the transition of inactive RAS-GDP to active RAS-GTP, enabling to transmit the proliferation and differentiation signals through its downstream effectors. Subsequently, the active RAS can be quickly deactivated by the action of GAPs. **c** RAS domain. The effector lobe (1–86 a.a.), allosteric lobe (87–165 a.a.), and HVR (167–188/189 a.a.) are all parts of the structure of RAS proteins. The effector lobe contains switches I (30–40 a.a.) and II (60–76 a.a.) are involved in effector binding and GEF or GAP binding, respectively. **d** RAF domain structure. RAF proteins consist of three conserved regions (CR1, CR2, and CR3) or two functional domains: an N-terminal regulatory domain and a C-terminal catalytic domain. **e** RAF dimerization. In the absence of cellular stimulation, RAF tends to exist in the monomeric, autoinhibited state. Upon stimulation by RAS-GTP, the autoinhibitory domain is released, freeing the inactive kinase domain to form homo- or heterodimers (with kinase suppressor of RAS [KSR]). Dimerization triggers mutual phosphorylation of the dimer components, fully activating the kinase. Phosphorylation and activation of target proteins, such as MEK1 and MEK2, propagates the MAPK cascade, leading to ERK1/ERK2 activation. This figure was created with BioRender.com

RAS/RAF/MAPK can be activate via two pathways: (1) a ligand-dependent pathway, in which ligands, such as growth factors, hormones, or cytokines, physically engage with receptors; and (2) a ligand-independent pathway, in which signaling is induced by a physical stressor, such as radiation, injury, or osmotic pressure.^59^ Aberrant RAF activation or mutations in upstream activators such as RAS or receptor tyrosine kinases (RTKs) can contribute to the development of malignancies in humans.^64^ Beyond RAS mutations, disruptions of RAS upstream components can also impact RAF activation. Receptors engaged by various growth factors, including TGF-α, EGF, VEGF, and platelet-derived growth factor-beta (PDGF-β), can instigate the canonical RAS–RAF–MEK–ERK pathway during various biological processes (Fig. 3a).^65–67^ Further investigations into RAF activation shed light on critical molecular mechanisms underlying cell proliferation, survival, and metastasis in cancer, particularly through the influence of the EGF receptor (EGFR) and small RAS-GTPases.^68^ Consequently, extensive research is currently underway to target RAF kinase as a promising avenue for the development of anticancer drugs.^68,69^

#### RAS

RAS, a pivotal upstream protein in the RAF/MAPK pathway, holds the distinction of being the founding member of the extensive RAS superfamily of small GTPases.^70^ RAS is extremely prevalent, as RAS mutations are detected in approximately 30% of all tumors. RAS activity varies across different cancer types. For example, NRAS is activated in lymphoid and myeloid malignancies, whereas KRAS is highly elevated in colon and pancreatic cancers, and HRAS activity is upregulated in bladder and kidney cancers. This upregulation of RAS activity within the context of cancer leads to the dysregulation of downstream protein kinase activities.

##### RAS structures and activations mechanism

RAS activation is triggered by various extracellular stimuli, with the primary mechanism involving the formation of complexes comprising autophosphorylated growth factor receptors, the adapter protein GRB2 and the exchange factor SOS.^71^ It has been proposed that RAS dimerization plays a critical role in facilitating RAS signal transmission, directing influencing RAF activation.^72^

In their normal and resting states, RAS proteins exist in an inactive GDP-bound form.^73^ Guanine nucleotide exchange factors (GEFs), like SOS, are recruited to the plasma membrane following the stimulation of mitogenic growth factors (Fig. 3b). Once GEFs bind to RAS, the stability of nucleotide binding disrupted, leading to the release of GDP from RAS and the transient formation of a nucleotide-free state. This, in turn, activates RAF and other downstream targets recruited by RAS-GTP. The signaling from RAS is terminated by the hydrolysis of GTP, which is mediated by the intrinsic enzymatic activity of RAS. Mammalian cells typically express three GEFs that are recognized as RAS activators: SOS, RASGRF, and RASGRP.^74^ Some RAS-related malignancies have been associated with GAPs, including NF1, p120GAP/RASA1, SynGAP/RASA5, GAP1 family, DAB2IP, and GAPVD1^74^. The conformational changes that accompany GTP hydrolysis are critical for RAS to function as a molecular switch in signaling pathways.^75^ There are four isomers of *RAS* genes, including *KRAS4A, KRAS4B, NRAS, and HRAS*. These isomers exhibit relatively consistent sequences or structures, encompassing N-terminal G-domains (1–166 a.a.) and C-terminal hypervariable regions (HVR) (166-189 a.a.).^76^ The G-domain conveys signals to downstream RAS effectors, featuring switch 1 (30–40 a.a.), switch 2 (60–76 a.a.), and a P loop (10–17a.a.) (Fig. 3c).^77,78^ The C-terminal region (final four amino acids, CAAX) undergoes posttranslational modification like iso-prenylation, proteolysis, and methylation, facilitating RAS localization and attachment to the membrane.^77^

#### RAF

RAF proteins, part of serine/threonine kinase family encoded by the *RAF* gene, serve as the upstream activator of MAPK and direct effector of RAS. In mammalian cells, there exist three distinct RAF proteins; ARAF, BRAF, and CRAF (also known as RAF-1). The initial member of the RAF family, CRAF, was initially characterized as an oncogene. Fusion of the CRAF catalytic domain with the retroviral Gag protein results in constitutive RAF kinase activation.^79^ Subsequently, two additional RAF family proteins, ARAF and BRAF, were discovered, each demonstrating similar function to that of CRAF.^32,80–82^ CRAF forms interactions with MEK, a dual-specificity kinase responsible for activating ERK.

##### RAF structure and activation mechanisms

The MAPK pathway is tightly regulated by several activation steps. The physical interaction between the RAF regulatory domain and membrane-bound RAS results in the attachment of RAF to the membrane, dephosphorylation, a conformation change for the kinase domain, and the subsequent phosphorylation of active sites (Ser338 and Tyr341).^83^ Many modulators mediate the negative or positive regulation of RAF activity through the formation of signaling complexes, which play critical roles in cancer growth and progression.^83,84^ To date, approximately 30 RAF-interacting proteins have been identified as putative RAF regulators.^83^

CRAF activation is a complex process in which Ser338, Tyr340, and Tyr341 are phosphorylated in response to oncogenes and growth factors. Many up- and downstream RAF effectors are associated with cancer transformation. Although the exact mechanisms underlying CRAF regulation remain unclear, phosphorylation of Ser338 and Tyr341 have been identified as crucial regulators of RAF kinase activity.^85^ In humans, all three RAF proteins are activated by phosphorylation at shared, conserved residues. Direct interaction between RAS and the N-terminal regulatory domain of CRAF is essential for RAF activation, and RAS mutations that cause constitutive RAF activation are detected in more than 30% of all human malignancies.^86–88^ However, RAS interaction alone is not sufficient to activate CRAF in vitro, indicating that other biochemical activities are necessary for RAF activation (Table 1). Some *RAS* mutants, including V12Y32F and V12T35S, are incapable of RAF activation but are able to interact with members of the RHO GTPase family.^13^ Previous studies have shown that p21-activated kinase (PAK) family members serve as molecular linkers, connecting RAS with RHO GTPases, including RAC and CDC42.^89^ In addition, CK2, JNK2, or SRC may be involved in CRAF activation through either RAS-dependent or RAS-independent mechanisms.^90–92^

**Table 1:** RAF-interacting activator proteins and regulatory mechanisms

RAF regulators		Regulatory mechanisms	Ref.
RAS	Direct activation	RAS plays an essential role in the activation of CRAF kinase, which is directly responsible for the activation of the MEK–ERK pathway.	^13^
		An undamaged CRAF zinc finger is necessary to bind to RAS and activate RAF in situ.	^85^
	Indirect activation	Dominant-negative Rac, Rho, and Cdc42 mutants prevent RAS-dependent transformation, whereas activated mutants work with CRAF to transform cells.	^573–578^
Type I PAKs (PAK1/2/3)	PAK1	PAK1 acts as a physiological candidate for CRAF phosphorylation on Ser338 during RAF activation.	^579,580^
	PAK2	Microtubule integrity regulates RAS-independent activation of CRAF through co-expression of small GTPases, including Rac, Cdc42, and PAK1/2.	^581^
	PAK3	PAK3 regulates CRAF activity by phosphorylating Ser338.	^582^
Type II PAKs (PAK4/5/6)	PAK4	PAK4 promotes premature senescence through a pathway including p16INK4/p19ARF and MAPK signaling.	^583^
	PAK5	PAK5 phosphorylates CRAF at Ser338, directing RAF to the mitochondria and contributing to anti-apoptotic action by phosphorylating BAD.	^582,584^
Other interacting proteins	CK2	CK2 acts as a component of the KSR1 scaffold complex during C/BRAF activation.	^167,585^
	Rac/Cdc42	Rac and Cdc42 act together with RAS and PI3K to achieve CRAF activation.	^586^
		Rac and Cdc42 induce CRAF activation with RAS.	^13^
	Src	Activated Src tyrosine kinase stimulates CRAF and MAPK.	^587^
		Src activates CRAF via RAS-independent pathways in vivo and in vitro.	^90^
		CNK1 regulates CRAF activation through Src.	^588^
	Hsp90	The Hsp90 and p50 (cdc37) complex regulates CRAF activity and stability.	^589,590^
	Cdc25A	Cdc25A regulates CRAF tyrosine phosphorylation.	^591^
	PKCs	Sequential activation of PKC isoforms (α and ε) contributes to CRAF and ERK1/2 activation.	^592^
	AKT	AKT physically interacts with BRAF and balances the cross-regulation between the PI3K–AKT and RAS–RAF–MEK signaling cascades.	^593^
		AKT3 collaborates with BRAF V600E, reducing activity to levels that favor cell proliferation rather than senescence.	^239^
	JAK	The Hopscotch JAK kinase requires the CRAF pathway to enhance blood cell activation and differentiation.	^594^
		JAK2, together with RAS and CRAF, activates ERK and MAPK in response to growth hormones.	^91^
	PP2A	PP2A functions as a CRAF-associated kinase activator involving the dephosphorylation of 14-3-3 binding sites in KSR and CRAF.	^595,596^

*AKT* protein kinase B, *BAD* BCL2-assocaited agonist of cell death, *CNK* connector enhancer of KSR, *CK2* casein kinase 2, *ERK* extracellular signal-related kinase, *JAK* Janus kinase, *KSR* kinase suppressor of RAS, *MAPK* mitogen-activated protein kinase, *MEK* MAPK kinase, *PAK* p21-activated kinase, *PI3K* phosphoinositide 3-kinase, *PKC* protein kinase C, *PP2A* protein phosphatase 2A

RAF proteins do not possess inherent subcellular localization motifs, and initially, they are within the cytoplasm in an inactive monomeric form.^93^ The activation of RAF, transitioning it from its autoinhibited, pre-signaling, and inactive state, necessitates a series of regulatory steps. These include the relief of autoinhibition, the formation of dimers or higher-order multimers, and phosphorylation.

##### Autoinhibition of the pre-signaling, inactive state

All RAF family members contain three conserved regions (CR1, CR2, and CR3) and two functional domains: an N-terminal regulatory domain and a C-terminal catalytic domain. The N-terminal regulatory domain contains both CR1, composed of a RAS-binding domain (RBD) and a cysteine-rich domain (CRD), and CR2, which is enriched in Ser/Thr residues, whereas CR3 is located at the C-terminal domain (Fig. 3d). RAF activation is largely accomplished through the removal of inhibitory enforcement at the RAF catalytic domain. The N-terminal regulatory region interacts with the kinase domain, leading to RAF autoinhibition, a fundamental regulatory mechanism shared by all three RAF proteins (ARAF, BRAF, and CRAF). However, both ARAF and CRAF require additional steps to achieve maximal activity, such as the phosphorylation of activating residues and the dephosphorylation of negative regulatory residues.^94^

##### Autoinhibition relief

In the absence of cellular stimuli, RAF proteins exist in a monomeric, autoinhibited, inactive form. Activation of the RAF kinase domain requires the relief of N-terminal autoinhibition, which is accomplished through a series of events, including a change in the subcellular localization, protein–protein interactions, lipid interactions, and regulatory phosphorylation.^95,96^ RAF activation first requires the translocation of RAF from the cytosol to the plasma membrane, which represents a vital step. Experiments have shown that retaining RAF on the plasma membrane results in the constitutive activation of RAF in a RAS-independent manner.^97,98^ The RAF RBD interacts with the GTP-bound RAS effector domain by adopting a conserved, ubiquitin-like structure,^99,100^ and RAS binding with the RAF CRD (zinc-coordinated structure) can relocate RAF to phosphatidylserines in the plasma membrane regardless whether RAS is bound to GTP.^101–104^ However, both the RBD and the CRD are involved in the full activation of RAF.

##### Dimerization and activating phosphorylation

RAS engagement on the membrane increases the phosphorylation of the RAF kinase domain and RAF dimerization. Recent work indicates that RAF dimerization is necessary for RAS-dependent RAF kinase activity and correlates with the pathogenic role of disease-associated mutant RAF, which displays strong intrinsic kinase activity.^105^ The formation of the side-by-side RAF dimer involves a structural association between the N- and C-terminal regions of the kinase domain.^106^ Following the release of inhibitory domain from the complex, the RAF kinase domain readily forms RAF–RAF homodimers, subsequently leading in kinase activation.^107^ The RAF-related pseudo-kinase KSR (kinase suppressor of RAS) also participates in forming side-to-side heterodimers with RAF (RAF-KSR heterodimer). Activated RAF kinase phosphorylates target proteins, such as MEK1 and MEK2, leading to the subsequent activation of ERK1 and ERK2 (Fig. 3e). By contrast, inhibitory phosphorylation of the RAF hinge region can disrupt and inactivate dimeric structures. Hyperphosphorylated RAF proteins are recycled to an inactive state, ready to receive a new round of activating signals.^108–110^

RAF and MEK1 activity can also be regulated by activated ERK via a feedback loop. Phosphorylation regulates the activities of RAF, MEK1, and ERK depending on the phosphorylation site.^111–113^ Upon signal engagement, active RAS promotes the exchange capacity of son of sevenless (SOS) through a positive feedback loop, eventually activating ERK1/2. By contrast, ERK-dependent SOS phosphorylation and disassociation of the SOS-Grb2 complex prevents RAS activation through a negative feedback loop.^114,115^ Therefore, the phosphorylation of target proteins in the ERK pathway can regulate associated signaling pathways based on the functional location of target proteins.^116,117^ Improved understanding of the RAS–RAF axis and RAF dimerization has revealed the roles played by RAF in many cellular conditions. In patients with cancer, RAF homo- and heterodimers likely mediate cellular responses to ATP-competitive inhibitors and cancer progression, suggesting that RAF dimers may represent potential therapeutic targets.

#### MEK and ERK

MEK represents a family of protein kinases that possess dual-specificity for tyrosine and serine/threonine residues, facilitating the activation of ERK by phosphorylating regulatory Tyr and Thr sites. Upon interaction of the catalytic VIII sub-region of RAF with MEK via its C-terminal catalytic domain, a serine residue becomes phosphorylated, thereby initiating MEK activation. The primary targets for phosphorylation by activated RAF are dual-specificity kinases, such as MEK1 and MEK2, with molecular weights of 44 and 45 kDa, respectively.^83^ Subsequent to MEK-dependent phosphorylation, ERK is set into action, triggering a range of functional responses in cells in response to growth factors or stressors. These responses are mediated by various cytoplasmic and nuclear substrates, including transcription factors.^118–120^

ERK (MAPK), a Ser/Thr protein kinase, occupies a crucial position in the cellular signal transduction network, and any aberrations in its activation faults can significantly impact cellular functions. When activated, MEK directly interacts with ERKs via its N-terminal region. In situations where multiple kinases are at work, they catalyze the bispecific phosphorylation of Tyr and Thr residues within the 8 “TEY box” of the sub-functional region of ERK, thereby activating ERK. Activated ERKs subsequently translocate to the nucleus, where they increase the phosphorylation of target proteins in the cytoplasm or regulate the activity of other protein kinases. This occurs before further phosphorylation and dimerization of ERK in response to signals that promote ERK activation.^121^

#### ERK downstream signals

Several ERK1/2 target proteins are ubiquitously found in cells.^60^ Including the cytoplasmic substrates death-associated protein kinase (DAPK), tuberous sclerosis complex 2, RSK, and MNK, and the nuclear transcription factor substrates nuclear factor of activated T cells (NF-AT), Elk-1, myocyte enhancer factor 2 (MEF2), c-Fos, c-Myc, and signal transducer and activator of transcription (STAT3). Some membrane-associated proteins (e.g., CD120a, Syk, and Calnexin) and cytoskeleton proteins (e.g., Neurofilaments and Paxillin) are also directly phosphorylated by ERK1/2.

### Other MAPK pathway

As summarized in Fig. 2, other several classical and atypical pathways and their related proteins are regulated by MAPKs.

#### The p38 signaling pathway

The p38 is reliably activated by a wide range of environmental stressors and inflammation and, in some cell types, by insulin and growth hormones. The p38 pathway is regulated by apoptosis-related receptors and physical sensors, including CDC42, RAC1, and mammalian Ste20-like kinases (MSTs), which also regulate JNK, resulting in the phosphorylation of the activation loop of MEK3/6. In particular, RAC1, a small G protein, controls the activation of p38 MAPK by a retinoic acid–induced beta1 integrin. p38 isoforms phosphorylate a wide range of cytoplasmic (e.g., cPLA2, MNK1/2, MK2/3, HuR, Bax, and Tau) and nuclear proteins (e.g., ATF1/2/6, MEF2, Elk-1, GADD153, Ets1, p53, and MSK1/2).^122^ p38 signaling is involved in immunological and inflammatory responses,^123^ cell fate determinants, and other stress responses^124^. Three anticancer compounds (e.g., SB203580, SB202190, and BIRB0796) specifically inhibit p38 isoforms (p38α and p38β) by competing with ATP in the binding pocket.^125^

#### The JNK pathway

The JNK pathway responds strongly to cytokines, growth factor deprivation, intracellular stimuli (e.g., DNA damage, cytoskeletal changes, oxidative, and ER stress), and extracellular stressors (e.g., UV radiation and osmotic stress). The JNK cascade is activated by adapter proteins in the TNF receptor-associated factor (TRAF) family, such as TRAF6, which is involved in the IL-1–induced activation of JNK.^126^ Two ATP-competitive JNK inhibitors, SP600125 (also known as JNK inhibitor II)^127^ and AS601245 (JNK inhibitor V),^128^ have been widely employed in the cancer research, although they exhibit low specificity. Many transcriptional factors (e.g., c-Jun, p53, ATF-2, NF-ATc1, Elk-1, HSF-1, STAT3, c-Myc, and JunB) are also regulated by JNK-directed phosphorylation.^129^ JNK plays an essential role in cell proliferation by modulating cell cycle genes^130^ and is involved in the differentiation of hematopoietic populations and the apoptotic response to cellular stressors.^131^

#### The ERK5 pathway

The ERK5 Pathway is activated by growth factors (e.g., epidermal growth factor [EGF], nerve growth factor, fibroblast growth factor [FGF]-2, and brain-derived neurotrophic factor); some cytokines, including leukemia inhibitory factor; and stressors, such as osmotic stress and hydrogen peroxide. ERK5 can be activated by several upstream factors, such as c-SRC, RAS, LAD1 adapter protein, and WNK Ser/Thr kinases^132^ In addition, ERK5 is activated by dual phosphorylation with a unique MAPK/ERK kinase 5 and MEK5,^133,134^ and activated ERK5 phosphorylates several cellular proteins, including the MEF2 transcription factor family, Sap1a (ETS domain transcription factor), c-MYC, serum and glucocorticoid inducible protein kinase, Connexin 43, and Bcl-2 agonist of cell death (BAD).^132^ Similar to ERK1/2, ERK5 is involved in cell survival and proliferation, increasing cyclin D1 expression during the G1/S transition.^135^ ERK5 is also necessary for vascular endothelial growth factor (VEGF)-mediated survival and tubular morphogenesis in primary human microvascular endothelial cells and the MEK inhibitors PD98059 and U0126 effectively inhibit ERK5.^136^

#### ERK3/4, ERK 7/8, and NLK

The ERK3/4 and ERK7 pathways are poorly characterized, although these proteins autophosphorylate the activating loop residues in vitro and in vivo.^137–139^ Serum and hydrogen peroxide stimulate ERK8 phosphorylation via conventional MAPKs. The MAPKAPK MK5 is the only known target of ERK3/4 according to several previous studies.^137,140–144^ ERK7/8 directly controls a number of proteins (e.g., Myelin basic protein [MBP], c-FOS, and c-MYC) in vitro, although their cellular functions are not clear.^139,145–147^ Many cytokines, including interleukin (IL)-6, granulocyte colony-stimulating factor, and transforming growth factor-beta (TGF-β), are associated with the activation of NLK, which is a key regulator of cell fate determination. NLK is triggered by Wnt pathway stimulation (Wnt-1 and Wnt-5a) and TGF-β.^148^ Last, NLK targets T-cell factor/lymphoid enhancer factor (TCF/LEF) transcription factors and STAT3.^148^ ERK3, ERK7, and NLK are involved in cell proliferation, cell cycle progression, migration, invasion, apoptosis, and cell differentiation. However, few inhibitors for atypical MAPKs have been validated as anticancer drugs, although GPLG0259, an inhibitor of MK5, is currently under clinical study for use in obesity and diabetes.

### Accessory proteins in the RAS/RAF/MAPK cascade

**Fig. 4:**
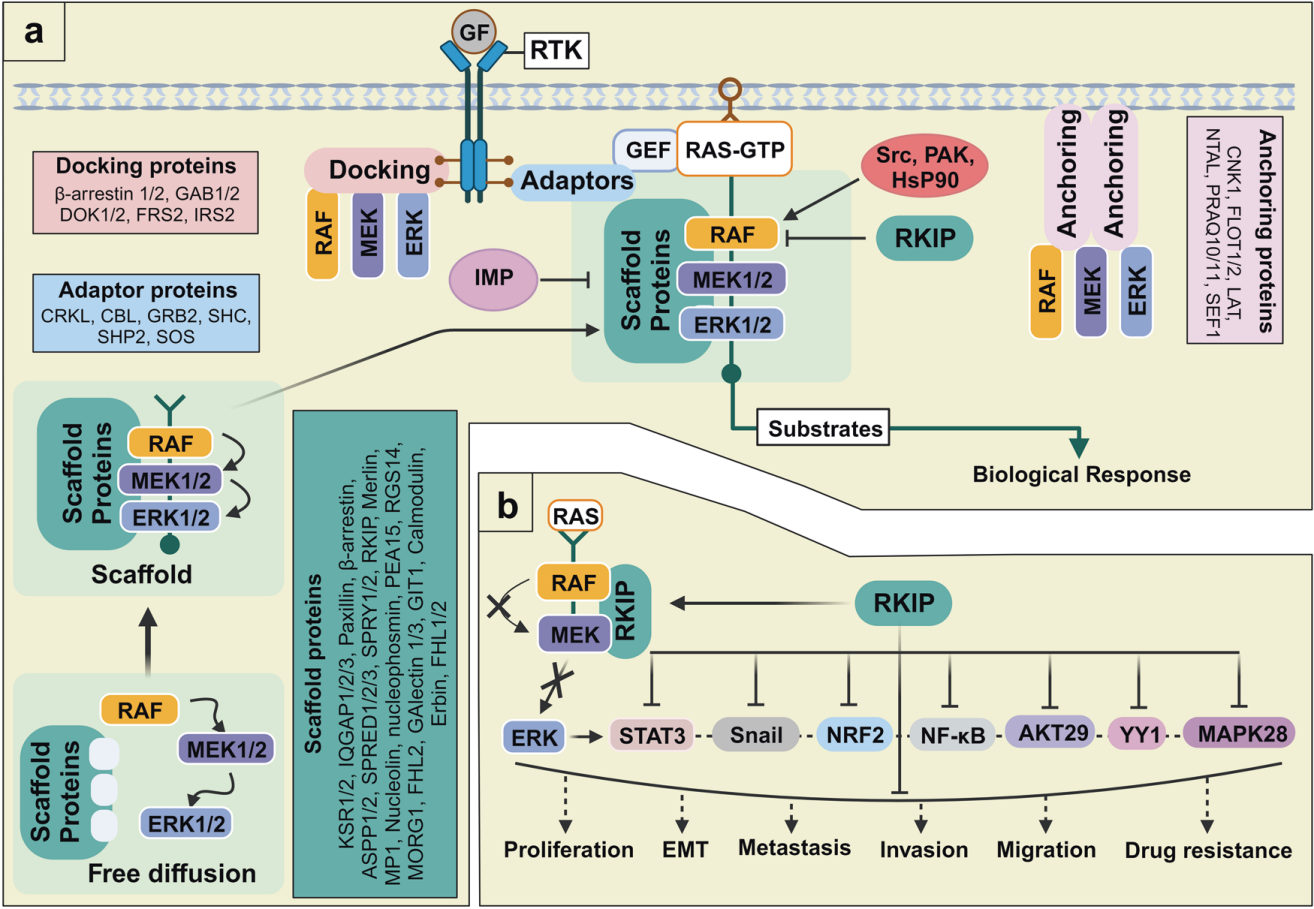
RAF signaling regulation by accessory proteins. **a** Accessory proteins consist of anchoring proteins, docking proteins, adapter proteins, and scaffold proteins. Anchoring proteins bind to membrane kinases and other effectors, whereas adapter proteins link receptor kinases with guanine exchange factors (GEFs). Docking proteins connect active receptors with multiple effectors. Scaffold proteins offer a signaling platform for the spatial regulation of the mitogen-activated protein kinase (MAPK) pathway. **b** CRAF kinase inhibitor protein (RKIP) is a tumor suppressor. RKIP, an intrinsic RAF kinase inhibitor, is associated with many malignant features, including metastasis and chemotherapy resistance, through the regulation of oncogenic mediators and signaling axes, such as NF-kB, YY1, MAPK28, STAT3, NRF2, and AKT29. Arrows and bars indicate stimulating and inhibiting signals, respectively. This figure was created with BioRender.com

MAPK signaling is linked to various malignancies in humans, and its activation is associated with many extracellular signals and intracellular proteins.^149^ Therefore, targeting the constituents of this signaling cascade frequently results in severe toxicity, activation of backup mechanisms, and reduced drug efficacy, often associated with an increase in therapy burden. To avoid these undesirable effects, other approaches targeting RAF modulators must be developed. The spatiotemporal characteristics of MAPK pathway constituents may offer an alternative strategy MAPK accessory proteins are spatially assembled to promote cooperation during signaling^150^ and can be divided into four categories: (1) anchoring proteins, (2) docking proteins, (3) adapter proteins, and (4) scaffold proteins (Fig. 4a).

#### Anchoring proteins

Anchoring proteins are bound to the membrane and connect and associate with effector proteins, which primarily consist of kinases. For example, the A-kinase-anchoring protein (AKAP-Lbc) and the scaffolding protein (KSR-1) constitute a signaling network that efficiently relays signals from RAF to MEK to ERK1/2.^151,152^ Many anchoring proteins have been discovered in other signaling pathways, including connector enhancer of KSR **(**CNK)1,^153^ Flotillin ½,^154^ linker for activation of T cells,^155^ non–T cell activation linker (NTAL),^156^ progestin and adipoQ receptor family members (PAQR10/11),^157^ and Sef1.^158^

#### RAF docking proteins

Docking proteins play a crucial role in cellular signaling by binding to receptors like RTKs and GPCRs, subsequently recruiting effector molecules. These specialized proteins typically possess PTB domains that enable them to selectively interaction with activated RTKs, as well as PH domains that serve to extend their presence at the cell membrane.^159^ The FGF receptor substrate 2 (FRS2) was initially identified as a substrate for the FGF receptor^160^ but was later found to be a key docking protein for various RTKs.^161^ Other identified docking proteins include docking protein 1/2 and GRB2-associated binding protein GAB1/2.^162^

#### RAF adapter proteins

Adapter proteins connect two functional components (e.g., receptor and GEF), providing additional docking sites for signaling proteins and promoting signal transduction from one-way receptors. Phosphorylation at Ser residues generates protein–protein interaction sites mediated by adapter proteins, such as the 14-3-3 family, facilitating associations with various signaling modulators, including CRAF, KSR, B-cell receptor (BCR), and phosphoinositide 3-kinase (PI3K).^163^ Many adapter proteins play important roles in signaling pathways, including CRK proto-oncogene (CRK and CRKL), Casitas B-lineage lymphoma, GRB2,^164^ SHC, SH2-containing protein tyrosine phosphatase 2 (SHP2) and SOS.^165^

#### RAF scaffolding proteins

Scaffolding proteins bind two or more partners to provide a signaling platform able to regulate the MAPK pathway both spatially and temporally.^166^ MAPK signaling components that exist as freely diffuse cytosolic forms are unable to effectively transmit signals to corresponding partners. Scaffolding proteins offer a platform at which many components can associate, allowing the efficient propagation of signals. Scaffolds also facilitate tighter control of MAPK signaling.^150^ KSR is a scaffold protein in the MAPK pathway that assembles B/CRAF, MEK1/2, and ERK1/2.^167^ In addition, both KSR and MEK partner-1 (MP1) retain ERK proteins in the proximity of critical cellular effectors.^168^ Several scaffolding proteins are involved in cellular functions^169^, including IQGAP1/2/3,^170^ Paxillin,^171^ β-arrestin,^172^ apoptosis stimulating proteins of p53 1/2 (ASPP1/2),^173^ SPRED1/2/3,^174^ SPRY1/2,^175^ RAF kinase inhibitory protein (RKIP), Merlin,^176^ Nucleolin and Nucleophosmin,^177,178^ PEA15,^179^ Regulator of G-protein signaling 14 (RGS14),^180^ MAPK organizer 1 (MORG1),^181^ Galectin 1/3,^182^ GIT1,^183^ Calmodulin,^184^ Erbin,^185^ and FHL1/2.^186^

RKIP is ubiquitously expressed in a broad range of cells and serves as an integral scaffolding protein^187^ and a negative modulator of the RAF–MEK–ERK signaling pathway^188^. RKIP directly binds CRAF and inhibits the MEK-dependent phosphorylation of CRAF by interfering with the formation of a kinase–substrate complex between CRAF and MEK.^189^

### RKIP as a tumor suppressor

RKIP belongs to the phosphatidylethanolamine-binding protein family, which functions in lipid metabolism and phospholipid membrane biogenesis.^190^ RKIP is a highly dynamic protein with a malleable pocket loop that exists in a variety of states, serving as a functional switch. This protein has pleiotropic roles in several signaling pathways involved in physiological processes As a MAPK signaling modulator, RKIP can inhibit the metastatic process by modulating RAF activation and may represent a new avenue for therapeutic intervention (Fig. 4b). RKIP also regulates cancer development and progression, and^191^ its expression is severely downregulated in many cancer tissues, including breast cancer^192^, prostate cancer^193^, gastric cancer,^194^ lung cancer,^195^ esophageal squamous cell carcinoma,^196^ colorectal cancer,^197,198^ and nasopharyngeal carcinoma.^199^ Low RKIP expression levels are generally associated with malignant features, such as metastasis and chemotherapy resistance, promoting oncogenic signaling axes, including nuclear factor kappa B (NF-κB),^200^ YY1,^201^ MAPK28,^202^ and AKT29.^203^ RKIP levels are regulated by STAT3 activation during metastasis in NSCLC cells,^204^ and RKIP downregulation leads to nuclear factor erythroid 2-related factor 2 (NRF2) hyperactivation, which is responsible for the development of chemotherapeutic resistance in colorectal cancer cells.^205^ Reduced RKIP levels stimulate invasion, metastasis, and radio-resistance in nasopharyngeal cancer cells.^206^

### Physiological functions of RAS/RAF/MAPK

The RAS/RAF/MAPK pathway involves signal transmission from membrane-based receptors, which interact with mitogens, to various destinations with cells, including the nuclear, cytoplasmic, and cell. These signals play a pivotal role in orchestrating a diverse range of physiological responses, encompassing cell proliferation, tumor invasion and metastasis, cellular metabolism, cell cycle progression, and ultimately cell survival or death.^207^ Consequently, any disruption or dysregulation of the RAS/RAF/MAPK pathway is closely linked to numerous human disorders, most notably cancer.

#### Role of ERK/MAPK in tumorigenesis

While tumorigenesis and the metastatic spread of cancer involve multiple cooperative cellular signals, the significance of the ERK/MAPK signaling pathway cannot be overstated when it comes to cancer invasion and metastasis. Notably, a heightened activation of ERK is evident across a spectrum of human cancer types, including ovarian, colon, breast, lung cancer, and others.^208^ Furthermore, in vitro experiments have revealed that microRNA-508 effectively inhibits EMT, migration, and invasion in ovarian cancer cells by modulating the ERK/MAPK1 signaling system.^208^ Meanwhile, in vivo studies have demonstrated that blocking the MAP kinase pathway leads to the suppression of growth of colon cancer cells.^68^ Additionally, Emodin has been found to inhibit the proliferation of non-small-cell lung cancer (NSCLC) cells by inducing PPARs and subsequently reducing Sp1 levels through the activation of ERK and AMPK.^209^

#### Cell proliferation and cell apoptosis

The ERK/MAPK signaling pathway primarily plays a role in promoting cell proliferation and exerts an anti-apoptotic influence. Specifically, under hypoxia conditions, it facilitates the survival of nutrient-starved tumor cell by reducing their susceptibility to apoptosis.^210^ Moreover, in the context of large B-lymphoma cells, microRNA-101 exerts control over cell proliferation and apoptosis by directly modulating MEK1 in the RAS/RAF/MAPK pathway.^211^ Furthermore, both ERK1 and ERK2 contribute to cell proliferation in a manner that depends on their expression levels, as they integrate signals from RAS, RAF, and MEK.^212^ The constitutive activation of the RAS/RAF/MAPK pathway contributes to tumorigenesis by inhibiting Caspase-9 through MAPK-dependent phosphorylation at Thr125.^213^

#### Cell cycle progression

Numerous downstream effectors of the RAS/RAF/MAPK pathway have a multifaceted impact. They not only drive cell cycle progression by instigating the production of cyclins and cell cycle-dependent protein kinases (CDKs) through the regulation of MYC and E2F but also orchestrate an early G1 cell cycle arrest. This arrest is achieved by influencing the expression of various CDK inhibitor proteins, including p16Ink4a, p15Ink4b, and p21Cip. Furthermore, the RAS/RAF/MAPK pathway is closely linked to the induction of cellular senescence, which is mediated by these CDK inhibitors, resulting in a premature G1 arrest.^214^

#### Tumor ECM Degradation and angiogenesis

Furthermore, the RAS/RAF/MAPK pathway plays a vital role in degradation of extracellular matrix proteins, a crucial process for cancer metastasis and angiogenesis. For instance, Mesothelin, a secretory protein, stimulates the production of MMP-7 by activating the MAPK/ERK and JNK signaling pathways in ovarian cancers^215^. Additionally, p70S6K, a downstream target of the RAS/RAF/MAPK pathway, exerts control over tumor growth and angiogenesis by promoting the activation of HIF-1alpha and the production of VEGF in ovarian cancer cells.^216^

It is fair to state that as we understand more about the specifics of RAF (ARAF, BRAF, and CRAF), we found that there are more unanswered questions about how they work and how they specifically affect physiological functions.

### Cell regulatory pathways mediated by MAPK-independent RAF kinase

**Fig. 5:**
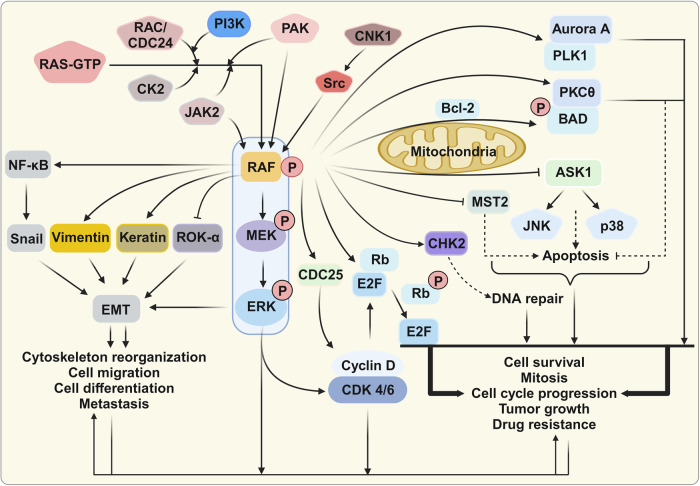
Kinase-independent regulation of RAF-interacting signaling. Three RAF effector proteins, Bcl-2 agonist of cell death (BAD), apoptosis signal-regulating kinase 1 (ASK1), and mammalian Ste20-like kinase 2 (MST2), are kinase-independent negative regulators of apoptosis. RAF can enhance cell cycle progression via the extracellular signal-related kinase (ERK) pathway, and RAF can regulate the cell cycle in a kinase-independent manner. RAF interacts with polo-like kinase 1 (PLK1) and Aurora kinase A (Aurora-A). During cell migration, RAF also functions as a spatial regulator of Rho-associated kinase (ROK)-α, a downstream effector of RHO, in a kinase-independent manner by inhibiting ROK-α activity. Several additional RAF substrates, such as NF-κB, Vimentin, Snail, and Keratin, are associated with cytoskeleton organization. This figure was created with BioRender.com

Generally, RAF activation leads to ERK1/2 activation via MEK1/2 phosphorylation^217^. MEK1/2 was thought to be the only RAF kinase substrate prior to the discovery of MEK1/2-independent RAF functions.^218,219^ Although only a few MEK-independent RAF targets have been defined, these MEK-independent RAF activities are thought to be important for carcinogenic regulation (Fig. 5).

#### RAF as an apoptosis regulator

MEK-independent RAF is a negative regulator of apoptosis. Three RAF effector proteins, BAD, apoptosis signal-regulating kinase 1 (ASK1), and MST2, play critical roles in the regulation of apoptosis. Interactions between RAF and these targets occur at the outer mitochondrial membrane, unlike classical RAF signaling, which is localized to the plasma membrane.^220^

BAD promotes apoptosis by inhibiting the pro-survival effects of BCL2 proteins,^221^ and RAF increases cell survival through the direct phosphorylation of BAD.^222^ Raf also acts as an adapter protein that stimulates the binding of BAD with protein kinase C, which phosphorylates BAD and inactivates downstream signals.^223^

ASK1, also referred to as MAP3K5, is a Ser/Thr kinase able to activate the SAPK, JNK, and p38 pathways to trigger apoptosis under oxidative conditions.^217,224^ FGF receptor activation increases interactions between RAF and ASK1 in the mitochondria, preventing the activation of the p38 MAPK pathway in an ERK- and PI3K-independent fashion.^225,226^ RAF modulates ASK1 activity, which is necessary for JNK- and p38-induced apoptosis, and the loss of RAF expression increases ASK1 activity, followed by increased JNK and p38 activation. *ASK1* knockout reverses the effects of RAF loss.^227^

RAF can inhibit the MST2-associated tumor suppression pathway in various cancers. RAF binds MST2 independent of kinase activity, preventing apoptosis in cancer cells via a two-pronged mechanism.^228–231^ First, RAF binding to MST2 blocks MST2 dimerization, a critical step for MST activation. Second, RAF recruitment of a phosphatase can prevent MST2 autophosphorylation at the Thr180 residue in the activation loop.^228,232^

#### RAF as a cell cycle and mitosis checkpoint mediator

CRAF can promote cell cycle progression in a MEK/ERK-independent manner^233^. CRAF directly interacts with key regulators of mitotic progression, polo-like kinase 1 (PLK1) and Aurora kinase A (Aurora-A).^233,234^ At the G2/M transition during mitosis, CRAF phosphorylation (Ser338) induces protein localization to centrosomes and mitotic spindle poles, where CRAF interacts with and activates Aurora-A and PLK1, leading to mitosis and tumor growth.

In addition, RAF regulates checkpoint kinase 2 (CHK2) activity during cell cycle progression. Upon DNA damage, PAK1 induces the formation of a RAF–CHK2 complex to stimulate the DNA repair system.^235^ RAF phosphorylation at Ser338 promotes the RAF–CHK2 interaction, which is associated with radiation resistance and CHK2 activation. In addition, RAF interacts with CDC25 phosphatase, which links mitogenic signaling with cell cycle activation.^236,237^

#### RAF as a regulator of the EMT and cytoskeletal organization

Cancer metastasis has been linked to the dysregulation of various signal pathways, including the constitutive activation of MAPK, NF-κB, and PI3K in melanoma.^238^ RAF-dependent AKT3 inhibition has also been associated with the promotion of cell proliferation, survival, and metastasis and the inhibition of cellular defense mechanisms and cellular senescence.^239,240^ NF-κB activation promotes metastasis by increasing the expression of many metastatic genes, including cyclooxygenase 2 (*COX2)*, genes encoding metalloproteinases, *VEGF*, and *SNAIL*.^241,242^ Jacqueline’s group demonstrated that RAS can activate NF-κB transcriptional activity via either RAF-dependent or RAF-independent mechanisms, both of which are dependent on SAPKs, such as p38.^243^ According to another study, RAF-dependent NF-κB activation involves MEKK1 rather than the traditional mitogenic cytoplasmic kinase pathway.^244^ RAF stimulates the expression of atrial natriuretic factor (ANF) in cardiac myocytes, whereas MEK1/2 inhibits ANF expression.^245^

RAF-dependent RHO signaling is related to cell migration. During cell migration, RAF functions as a spatial regulator of RHO-associated kinase (ROK)-α, an effector downstream of RHO, in a kinase-independent manner.^246^ In a conditional knockout study, RAF was found to be necessary for proper wound healing in vivo and for keratinocyte and fibroblast cell migration in vitro. This study also indicated that RAF-mediated ROK-α inhibition is necessary for RAS-dependent carcinogenesis.^247^ Functionally, the interaction between RAF and ROK-α may be associated with a RAF-induced anti-apoptotic signal, as stimulation of the FAS death receptor increases RAF–ROK-α complex formation.^248^ In addition, ROK-α kinase activity is inhibited by the RAF regulatory domain.^249^ However, ROK-α is likely not regulated by RAF in *RAS*-mutant tumors.^250^ Moreover, several additional RAF substrates are involved in cytoskeleton organization, including Vimentin^251^ and Keratin 8.^252^

### Crosstalk between RAS/RAF/MAPK and PI3K/AKT/mTOR or MST2/Hippo signaling pathways

**Fig. 6:**
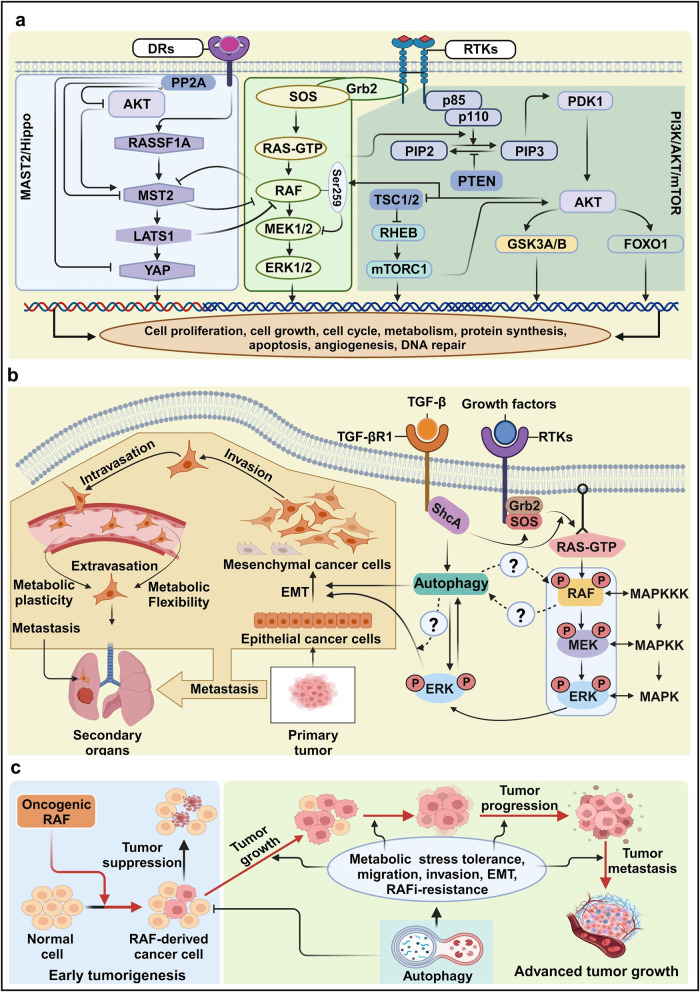
The crosstalk of RAS/RAF/MAPK with other pathways. **a** Crosstalk between the RAS/RAF/MAPK, PI3K/mTOR or MAT2/Hippo signaling pathways. The RAS/RAF/MAPK signal collaborates within its own cascade and interfaces with the PI3K/mTOR pathway, where its influence is MAPK-dependent. Conversely, the MST-2/Hippo pathway operates independently of MAPK activity but relies on the presence of RAF for its functionality. **b** Functional interactions between autophagy and mitogen-activated protein kinase (MAPK) signaling during the epithelial-to-mesenchymal transition (EMT). The MAPK pathway can be activated by canonical receptor tyrosine kinases (RTKs) and through Smad-independent activation by transforming growth factor-beta (TGF-β). Both signals activate a typical RAS–RAF–MAPK cascade, stimulating the EMT process. During the metastasis process, cancer cells migrate from a primary site to a secondary site facing many stressors, and therefore metabolic and cellular alterations are necessary to overcome these stressors. **c** The role of autophagy in tumorigenesis. During early tumorigenesis, autophagy acts as a tumor suppressor. However, autophagy drives tumor growth, progression, and metastasis by enhancing migration, invasion, EMT, and metabolic tolerance during advanced stages of cancer, allowing cancer cells to evade RAFi therapy. Arrows and bars indicate stimulating and inhibiting signals, respectively. This figure was created with BioRender.com

The PI3K-mTOR (mammalian target of rapamycin) pathway is a crucial mechanism governing cell survival, division, and metabolism. Growth factors can engage the pathway by either directly recruiting PI3K to their receptors or indirectly involving it through docking proteins like IRS (insulin receptor substrate) or GAB (GRB2-associated binder). This activation of PI3K leads to the generation of the secondary messenger phosphatidyl inositol 3,4,5-triphosphate (PIP3), which in turn recruits the protein kinase AKT to the plasma membrane. Subsequent AKT activation, dependent on PDK1, initiates the phosphorylation of numerous factors related to survival, proliferation, motility, and the TSC2 GAP (GTPase Activating Protein). AKT-dependent TSC2 phosphorylation releases TSC1/2 inhibition by the GTPase RHEB (RAS homolog abundant in brain), ultimately activating mTORC1, which regulates cell growth. Both the RAS/RAF/MAPK and PI3K/AKT/mTOR pathways frequently experience dysregulation in many human cancers, often due to genetic alterations in their components or upstream regulators.^253^ The intricate network of positive feedforward and negative feedback loops in these pathways significantly influences signal dynamics. Notably, the GAB docking proteins, forming the GRB2-SOS complex upon activation of RTKs, are key players in positive loops. This complex, which includes RAS-GAP, SHP2, PI3K, and PIP3 (a protein-tyrosine phosphatase with a Src homology two domain), contributes to RAS activation. Dephosphorylation of RAS-GAP docking sites on GAB1 by SHP2 reduces RAS activation and enhances RAS-ERK signaling. Additionally, GAB2-mediated PI3K recruitment generates local PIP3, further stimulating PI3K signaling. Furthermore, SOS, RAF, and MEK1 can be phosphorylated by ERK, which creates a negative feedback loop by dampening ERK activity (Fig. 6a). Indeed, comprehensive cancer genome analyses have revealed that over-activation or mutations that enhance the MAPK and PI3K pathways are characteristic features of many human cancer types. Importantly, the interplay between RAS/RAF/MAPK and PI3K/AKT pathways is tightly controlled in response to ligands in a dose-dependent manner. For Instance, elevated levels of insulin-like growth factor I (IGF-I) induce a rapid and potent phosphorylation of AKT at the serine 259 residue, effectively restraining RAF kinase activity. Conversely, low concentration of IGH-1 fail to induce such crosstalk, but still exert mitogenic effects^254^

The MST2 protein family, characterized by serine/threonine kinases, becomes activated in response to stress signals in mammalian cells, and their overexpression has been observed to trigger apoptosis.^232^ The MST2/Hippo pathway is intricately linked to the RAS/RAF/MAPK pathway **(**Fig. 6a**)**. Specifically, the MST-CRAF complex, induced by mitogenic and apoptotic signals, acts as a safeguard against unchecked cell proliferation.^229^ A previous study has shed light on the dynamic changes in protein-protein interactions (PPIs) arising from the function association between the kinases MST2 and CRAF kinases, along with the modulation of their respective upstream activators RASSF1A and RAS^231^. The interaction between CRAF and MST2 inhibits the binding of the scaffold protein RASSF1A to MST2, leading to MST2 dimerization and activation. Interestingly, activated AKT promotes the binding of MST2 to RAF and subsequently preventing MST2 activation.^255^ Additionally, MST2 can be inhibited by a phosphatase, likely PP2A, associated with RAF.^256^ Conversely, MST2 interferes with RAS-dependent RAF activation by blocking the RAS-binding domain (RBD) domain in CRAF. RASSF1A can rerelease MST2 from its inhibitory complex with CRAF, activating LATS1. This activation of LATS1, in turn, induces the formation of the YAP1-p73 transcriptional complex, ultimately leading to apoptosis.^257^ Furthermore, the RAS/RAF/MAPK pathway engages in crosstalk with several other signal pathways, including those involved in DNA repair process or cell cycle regulation, as depicted in Fig. 5.

### The link between autophagy and RAF signaling in cancer metastasis

Autophagy is an essential process that maintains cellular homeostasis via the lysosome-dependent degradation of cellular components, such as proteins and organelles, allowing cells to recycle macromolecules^258,259^. Irregular autophagy activation can lead to cellular dysfunction, as observed in many human diseases, including neurodegenerative diseases, heart disease, infectious diseases intervertebral disc degeneration, liver disorders, and cancers.^260–262^ Autophagy is also involved in various stress responses, developmental processes, and aging.^263,264^

Although autophagy can be either tumor-suppressive or tumor-promoting, depending on the context, the precise mechanisms that determine the role of autophagy are not well-defined. In general, increased catabolism driven by autophagy promotes cell survival, and autophagic abnormalities induce cell death in cancer cells. Cancer cells exhibit more microenvironmental and metabolic dependencies than normal cells, and targeting the double-edged process of autophagy represents an appealing option for the development of future therapeutic agents.^265^

The MAPK pathway drives the expression of autophagy-related-8 (Atg8) in cancer cells.^266^ Studies of *RAF* mutations suggest a functional association between autophagy and V600E-mutant BRAF. Autophagy accelerates the growth of V600E-mutant melanoma in mice by allowing the bypass of the senescence process.^267^ The reduced expression of autophagy-related *ATG* genes in patients with *BRAF*^*V600E*^*-*mutant melanoma appears to suppress carcinogenesis.^268^

Cancers can spread to secondary organs through a complicated, efficient, and deadly process called metastasis, which represents a substantial contributor to mortality.^269^ Metastasis requires coordination between the genetic programs that promote and prevent metastasis and the tumor microenvironment to allow cancer cells to transition from their initial locations and develop in secondary organs. Metastasis suppressor genes prevent metastasis at secondary sites with no impact on the primary tumor.

During metastasis, cancer cells dissociate from the primary sites, travel through the circulation, and deposit in a secondary site, overcoming many obstacles, including nutrient limitations and cellular stress.^270^ In metastatic cells, cellular metabolism undergoes dynamic alterations that promote a shift toward the metastatic cascade.^271^ Metabolic plasticity and flexibility in cancer cells play important roles in the metastatic process.^272^ Metabolic plasticity allows a single metabolite to meet multiple metabolic needs during metastasis, whereas metabolic flexibility allows several metabolites to fulfill the same metabolic need.^273,274^ The metabolites sapienate, generated by fatty acid desaturase 2, and palmitoleate, generated by stearoyl-CoA desaturase-1, are mono-unsaturated fatty acids involved in the metabolic flexibility of primary tumors.^275,276^ The presence of these metabolites in the bloodstream is essential for the invasion, migration, and survival of metastatic cells. These metabolites are also associated with the EMT,^277^ which represents a necessary step through which cancer cells acquire metastatic properties and plays important roles in cancer progression, metastasis, and drug resistance.^278^

Based on previous studies, MAPK signaling, autophagy, and EMT are physically and functionally interconnected during cancer metastasis (Fig. 6b). EMT is a multidimensional process that involves the remodeling of the cytoskeleton, cell membrane, and cell–cell junctions, resulting in the loss of epithelial characteristics and the acquisition of mesenchymal properties facilitated by MAPK activation.^279^ Autophagy supplies the energy required for TGF-β–induced EMT and cancer metastasis. TGF-β is involved in many cellular processes, including tissue fibrosis, growth inhibition, and EMT. TGF-β activates Smad-dependent and Smad-independent signaling pathways, including the ERK1/2 pathway, which is involved in cytoskeletal organization, cell growth, survival, migration, and invasion.^280^ TGF-β–directed MAPK signaling also activates common downstream signaling molecules induced by RTKs. In response to TGF-β, RAS triggers ERK1 and ERK2 activation, leading to the activation of RAF and MEK1/2, as shown in Fig. 2.^281,282^ Following TGF-β-induced Ser or tyrosine phosphorylation of the type I TGF-β receptor, ShcA recruits GRB2 and SOS to activate ERK1/2 through RAS, RAF, and MEK1/2.^281,283^ Other signaling molecules, such as integrin, Notch, Wnt, TNF-α, long non-coding (lnc) RNA, and EGF, synergize with TGF-β signaling to promote tumor invasion and metastasis.^284–290^ Many studies suggest the existence of functional connections between TGF-β and RAS–RAF signaling in tumorigenesis. TGF-β–induced EMT is enhanced by increased RAS–ERK^291^ signaling, and MEK1/2 pharmacological inhibition prevents TGF-β–induced EMT.^292^

EMT and autophagy are connected through multiple pathways, although the exact role of autophagy in EMT remains unclear. Autophagy inhibition promotes EMT, invasion, and metastasis in many cancer cells, including gastric, colorectal, melanoma, and pancreatic cancer cells, mouse embryonic fibroblasts, and keratinocytes.^293–295^ By contrast, TGF-β–induced autophagy promotes cancer cell migration via MAPK–ERK activation in NSCLC and SMAD4-negative pancreatic cancer cells.^296,297^ However, autophagy inhibits metastasis in HCC^298^ and prevents EMT in breast and SMAD4-positive pancreatic cancer cells.^297,299^

Autophagy plays a significant role in RAF-driven tumorigenesis, functioning as a tumor suppressor during early stages and as a tumor promotor during advanced stages (Fig. 6c). BRAF and CRAF activate autophagy to promote tumor cell survival.^27^ *Atg7*-knockout mice with *RAF*-mutant melanoma display reduced tumor growth and significantly increased survival compared to wild-type mice.^267^ Interestingly, autophagy exhibits both tumor-promoting and tumor-suppressive roles in the same mouse model of *BRAF*^V600E^–mutant lung cancer.^300^ However, autophagy promotes tumor growth and metabolism in *BRAF*^V600E^–mutant lung cancer,^301^ and *BRAF*^V600E^–mutant cancers promote autophagy to maintain mitochondrial function and glutamine metabolism.^302^

The fact that human cancer frequently exhibits dysregulation of the autophagy and RAS/RAF/MAPK pathways makes the components of these signaling cascades intriguing candidates for therapeutic intervention. Recent research has shown the existence of positive and negative feedback loops in these pathways, which activate one pathway when the other signaling cascade is blocked. Therefore, blocking both pathways with a combination of signaling inhibitors may have a stronger antitumor effect than using a single medication.

## Targeting the RAS/RAF/MAPK pathway for cancer therapy

Our understanding of the roles played by RAS/RAF/MAPK components in both normal physiology and pathological conditions has significantly progressed. In the realm of therapeutic interventions, RAS/RAF/MAPK inhibitors have emerged as promising targets for addressing BRAF-mutated cancers and other disorders. These inhibitors have gained approval from the FDA and are employed either as standalone treatments or in combination with two or more agents. These FDA-approved RAS/RAF/MAPK-targeted medications, including their most up-to-date information, are represented in Table 2 in the latest medication guide available at www.accessdata.fda.gov.

**Table 2:** FDA-approved Ras/Raf/MAPK targeting drugs and their limitations

Strategy/Targets	Drugs (Trade name) NDA/BLA	Company/Approval date	Indications/Biomarkers	Limitations	
				Common adverse effects (AD)/Incidence (%)	Remarks
Single therapy					
RAS	Adagrasib (KrazatiI) NDA 216340	Mirati Theraps Dec 12, 2022	NSCLC/KRas^G12C^	Nausea, diarrhea, vomiting, fatigue, dyspnea musculoskeletal pain, hepatotoxicity, renal impairment, and decreased appetite. ≥25%	The pharmacokinetics (PK) of adagrasib is constrained by CYP3A and ABCB1
	Cetuximab (Erbitux) BLA 125084	Imclone Feb 12, 2004	CRC, HNC/KRAS^WT^, EGFR	Fever, sepsis, kidney failure, dehydration, skin drying, fissuring, blepharitis, cheilitis, cellulitis, pulmonary embolus, and cyst. ≥25%	Approved and effective only in KRAS wild-type patients
	Panitumumab (Vectibix) BLA 125147	Amgen Inc Sept. 27, 2006	CRC/KRAS^WT^, NRAS^WT^	Dermatitis acneiform, pruritus, erythema, rash, skin exfoliation, paronychia, dry skin, and skin fissures. ≥20%	Approved and effective only in KRAS wild-type patients
	Sotorasib (Lumakras) NDA 214665	Amgen Inc May 28, 2021	NSCLC/KRAS^G12C^	Diarrhea, musculoskeletal pain, nausea, fatigue, hepatotoxicity, and cough. ≥20%	Oral availability and brain accumulation of sotorasib were limited by CYP3A and ABCB1, respectively
RAF	Vemurafenib (Zelboraf) NDA 202429	Hoffmann- La Roche Aug 17, 2011	Melanoma/BRaf ^V600E^	Arthralgia, maculopapular rash, alopecia fatigue change in the heart’s electrical activity, and skin growth (papilloma). ≥10%	Zelboraf has not been studied in patients with BRAF^V600^-negative, it blocks certain enzymes that promote cell growth
	Dabrafenib (Tafinlar) NDA 202806	Novartis May 29, 2013	Melanoma/BRAF^V600E^BRAF^V600K^	Hyperkeratosis, headache pyrexia, arthralgia, rash, papilloma, alopecia, palmar-plantar, back pain, erythrodysesthesia, cough, constipation, myalgia, and nasopharyngitis. ≥10%	Tafinlar is not indicated for the treatment of patients with wild-type BRAF melanoma.
	Encorafenib (Braftovi) NDA 210496	Array Biopharma Inc Jue 27, 2018	Melanoma, CRC/BRAF^V600E^ BRAF^V600K^	Hyperkeratosis, alopecia, PPES, fatigue, rash, arthralgia, dry skin, nausea, myalgia, headache, vomiting and pruritus. ≥25%	Braftovi can cause tumor promotion in BRAF wild-type tumors, new primary malignancies, fetal harm, impaired fertility, uveitis, hemorrhage
MEK	Trametinib (Mekinist) NDA 204114	Novartis May 29, 2013	Melanoma/BRAF^V600E^ BRAF^V600K^	Rash, diarrhea, lymphedema, stomatitis, hypertension, abdominal pain, hemorrhage, dry skin, pruritis, and paronychia. ≥10%	Mekinist is not indicated for the treatment of patients who have received a prior BRAFi therapy
	Cobimetinib (Cotellic) NDA 206192	Genentech Nov 10, 2015	Melanoma/BRAF^V600E^ BRAF^V600K^	Diarrhea, sensitivity to ultraviolet (UV) light (photosensitivity reaction), nausea, fever (pyrexia) and vomiting. ≥20%	Coadministration of colic with itraconazole (increased cobimetinib systemic exposure by 6.7-fold
	Selumetinib (Koselugo) NDA 213756	Astrazeneca April 10, 2020	PN/NF1	Vomiting, rash, abdominal pain, diarrhea, nausea, dry skin, fatigue, musculoskeletal pain, pyrexia, acneiform rash, stomatitis, headache, paronychia, and pruritus. ≥40%	It is not known if Koselugo is safe and effective in children under 2 years of age
Combination therapy					
RAF and MEK	Vmurafenib+cobimetinibe NDA 206192	Genentech Nov 1, 2022	Melanoma, histiocytic neoplasm/BRAF^V600E^ BRAF ^V600K^	Hyperkeratosis, headache, pyrexia, arthralgia, rash, papilloma, alopecia, palmar-plantar, back pain, erythrodysesthesia, cough, constipation, myalgia, and nasopharyngitis.10%	*Tafinlar is not indicated for the treatment of patients with wild-type BRAF melanoma*
	Dabrafenib + trametinib	Novartis Jun 22, 2017 April 30, 2018 June 22, 2022 Mar 16, 2023	Solid cancers, NSCLC, melanoma with involvement of lymph node(s), LGG/BRAF^V600E^ BRAF^V600K^	Pyrexia, fatigue, nausea, vomiting, diarrhea, dry skin, decreased appetite, edema, rash, chills, hemorrhage, cough headache, dyspnea arthralgia, myalgia, dyspnea, musculoskeletal pain, abdominal pain, dermatitis acneiform, dizziness, upper respiratory tract infection and weight increased. ≥15%	Tumor promotion in wild-type BRAF, resistance to BRAFi, *melanoma, hemolytic anemia, hyperglycemia, erythema, serious febrile drug reactions, uveitis, and iritis, RPED, RVO, cardiomyopathy, venous thromboembolic events, hemorrhage, and embryofoetal toxicity*
RAF and RAS	Encorafenib+cetuximab	Array Biopharma Inc April 8, 2020	CRC/BRAF^V600E^	Fatigue, nausea, diarrhea, dermatitis acneiform, abdominal pain, decreased appetite, arthralgia, and rash. ≥25%	Around 10% of patients who received Braftovi in combination with cetuximab had pancreatitis
PDL-1, RAF and MEK	Atezolizumab (Tecentriq) +vemurafenib or cobimetinib	Genentech July 30, 2020	Melanoma/BRAF^V600E^	Rash, musculoskeletal pain, fatigue, hepatotoxicity, pyrexia, nausea, pruritus, edema, stomatitis, hypothyroidism, and photosensitivity reaction. ≥20%	Tecentriq may cause fertility problems in females. The safety and effectiveness in children are unknown

*FDA* US food and drug administration, *NDA* new drug application, *BLA* biological license application, *NSCLC* non-small cancer lung cell, *CRC* colorectal cancer, *HNC* head and neck cancer, *LGG* low grade gliomas, *PN* plexiform neurofibromas, *NF1* Nnurofibromatosis type 1, *RPED* retinal pigment epithelial detachment, *PPE* palmar-plantar erythrodysesthesia, *RVO* retinal vein occlusion, *UV* ultraviolet, *≥* greater or equal, *%* percentage

### RAS/RAF/MAPK inhibitors

**Fig. 7:**
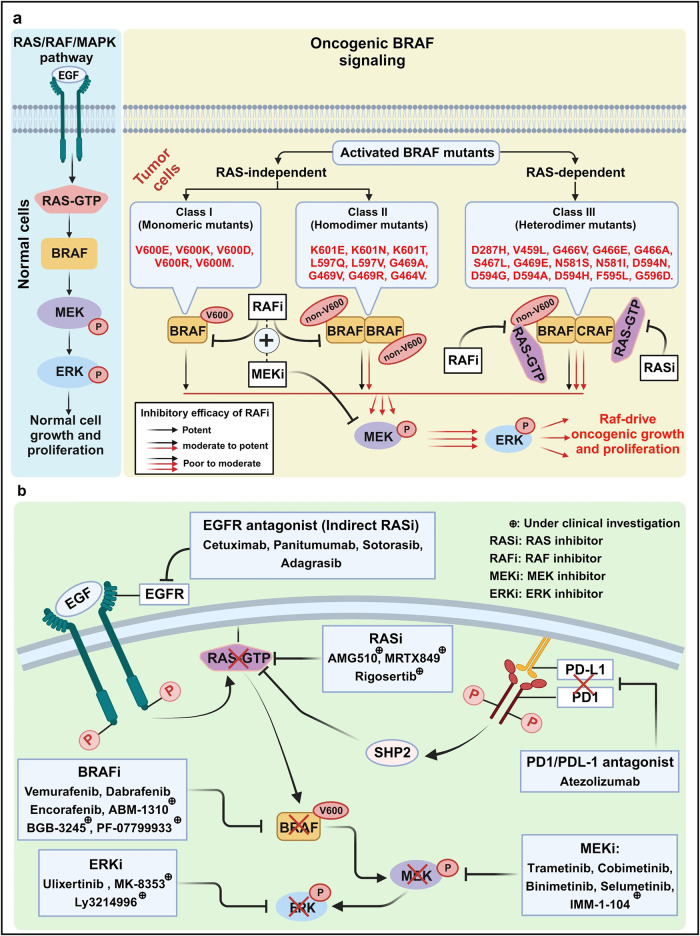
RAS/RAF/MAPK-targeted therapy in BRAF-mutated malignancies. **a** Functional classifications of *BRAF* mutations. In Class I, *BRAF* mutants (e.g., V600E) transmit signals via a monomeric form independent of RAS activation, leading to increased extracellular signal-related kinase (ERK) activation. In Class II, *BRAF* mutants (e.g., K601E) are RAS-independent, forming mutant–mutant BRAF dimers. RAF inhibitors (RAFi), such as Vemurafenib and Dabrafenib, block both Class I and II RAF kinases. In Class III, mutant BRAF (e.g., D287H, V459L) exhibits increased RAS binding and heterodimer formation between mutant BRAF and wild-type CRAF. MEK inhibitors (MEKi), such as Trametinib or Cobimetinib, show an additive effect when combined with RAFi for cancer treatment. **b** RAS/RAF/MAPK-targeted therapies. Specific Inhibitors targeting the RAS/RAF/MAPK pathway represent as each group of action: EGFR agonists (EGFRi), RAS inhibitors (RASi), RAF inhibitors (RAFi), MEK inhibitors (MEKi), and ERK inhibitors (ERKi). Arrows and bars indicate stimulating and inhibiting signals, respectively. This figure was created with BioRender.com

RAS/Raf/MAPK inhibitors represent a category of precision therapies employed in the management of diverse cancers, especially those marked by mutations in the RAS/RAF/MAPK pathway (Fig. 7a). These pathway constituents play a critical role in regulating cell proliferation, differentiation, and survival. However, when mutations disrupt their normal function, they can contribute to the initiation and progression of cancer.

#### KRAS-targeted therapy

The proto-oncogene *KRAS* plays a crucial role in cell signaling pathways governing cell growth and differentiation. In various cancers, mutations in the *KRAS* gene have been linked to poor prognosis and a limited range of targeted treatment options. In Western nations, lung adenocarcinomas exhibit *KRAS* mutations in 20–40% of cases, while the prevalence is slightly lower, around, in Asian countries.^303^ *KRAS* mutations pose therapeutic challenges as they are often associated with resistance to specific target therapies, such as epidermal growth factor receptor (EGFR) inhibitors in non-small cell lung cancer (NSCLC).^304^ While the development of the direct RAS inhibitors has been proven challenging due to the nature of RAS protein, ongoing research and clinical trials are exploring strategies and therapies to effectively target *KRAS*-mutant tumors.

##### Cetuximab and Panitumumab

Cetuximab and Panitumumab are monoclonal antibody therapies that target the epidermal growth factor receptor (EGFR) and are employed in the treatment of various tumors, including head and neck and colorectal cancer.^305^ Importantly, these drugs are approved and effective exclusively in *KRAS* wild-type patients with advanced colorectal cancer.^306^ While they are not direct RAS-mutant targeted therapeutics, they may occasionally have an indirect impact on RAS signaling pathways.^307^ For instance, when combined with Cetuximab, LSN3074753 (a pan-RAFi) demonstrates synergistic anticancer efficacy in *BRAF* or *KRAS*-mutant CRC PDX models.^308^ It has been suggested that colorectal cancers with the *KRAS*^*G13D*^ mutation may respond more favorably to Cetuximab or Panitumumab treatment compared to other more prevalent *KRAS* mutations.^309^ In the realm of KRAS-mutant targeted therapeutics, Adagrasib (MRTX849) demonstrated favorable tolerability and exhibited anticancer activity in patients with advanced solid tumors harboring the *KRASG12C* mutation in a first-in-human phase I/IB clinical trial (KRYSTAL-1) (*NCT03785249*).^310^

##### Sotorasib

The U.S. FDA-approved Sotorasib (Lumakras^TM^, Amgen) has been employed in the treatment of advanced NSCLC patients with and *KRAS*^*G12C*^ mutation who have undergone at least one prior systemic therapy.^311^ The FDA’s rapid approval of Sotorasib serves as a remarkable example of recent expeditious approvals for clinically effective drugs. In phase I clinical trials, Sotorasib demonstrated promising anticancer activity in heavily pretreated patients with advanced solid tumors bearing the *KRAS*^*G12C*^ mutation (*NCT03600883*).^312^ Subsequently, in phase II clinical trials, Sotorasib therapy provided sustained clinical benefit without revealing new safety concerns in previously treated KRASG^*12C*^-mutated NSCLC patients (*NCT03600883*). It is noteworthy that Sotorasib’s oral availability is significantly restricted by CYP3A, while its brain accumulation is robustly constrained by ABCB1.^313^

##### Adagrasib

Adagrasib (KRAZATI^TM^, Mirati Therapeutics) is an orally administered, and highly effective small molecule inhibitor, irreversibly covalent binding to KRAS. It have been developed for the treatment of solid tumors harboring the *KRAS*^*G12C*^ oncogenic driver mutation, including NSCLC and CRC.^314^ Tian et al. unequivocally demonstrates the promising effectiveness and acceptable safety profile of Adagrasib based on multiple registered interventional clinical trials (e.g., *NCT05375994* and *NCT05472623*) in patients with KRAS^G12C^-mutated NSCLC.^315^ They have also recommended further research to explore Adagrasib’s potential in various contexts and combination therapies. The US FDA has granted approval for expanded access to Adagrasib (MRTX849) in patients with advanced solid tumors carrying the KRAS^G12C^ mutation (NCT05162443), citing data from several interventional clinical trials (e.g., *NCT04975256*, *NCT05853575*, *NCT03785249*, *NCT04330664*, and *NCT05609578*). A recent phase I/II study showcased Adagrasib’s antitumor activity in heavily pretreated patients with metastatic CRC bearing mutant *KRAS*^*G12C*^, both as oral monotherapy and in combination with Cetuximab (*NCT03785249*).^316^ The pharmacokinetics (PK) of Adagrasib is constrained by the CYP3A and ABCB1 activity, and it can be modified by mouse plasma carboxylesterase 1 c.^317^

#### BRAF-mutant targeted therapy

##### RAF inhibitors (RAFi)

Mutations of RAF are frequently found in melanoma and other cancers. The prevailing mutation of RAF is BRAF^V600E^. Abnormal activation of the RAS/RAF/MAPK pathway, a hallmark of cancer, often originates from genetic alterations in *RAF*-encoding genes or RAF upstream genes. Consequently, the RAF kinase family is a promising target for potential cancer therapies. The RAF kinase activity is frequently disrupted in human cancer due to RAF itself or mutations affecting the upstream regulators and downstream effectors proteins as depicted in Fig. 7b.

PLX4720, a potent and selective RAF inhibitor, has demonstrated robust antitumor activity against *RAF*-mutant melanoma in vitro and in vivo.^318^ Vemurafenib (PLX4032), an analog of PLX4720, exhibits improved pharmacokinetic properties compared to PLX4720, and has received approval for the treatment of advanced melanomas and other cancers harboring *RAF* mutations.^319,320^ Vemurafenib has shown exceptional efficacy with manageable side effects, both as a monotherapy and when combined with MEK inhibitors, for patients with *RAF* mutant cancer. Dabrafenib is another effective therapeutic option for melanomas bearing the V600E *BRAF* mutation, achieving tumor shrinkage in over 90% of patients with half experiencing partial to complete responses.^321,322^ Furthermore, clinical phase III studies have demonstrated that Encorafenib, a BRAF inhibitor, either standalone treatment or in combination with MEK inhibitors, significantly prolongs progression-free survival compared to Vemurafenib.^323^ In previous studies involving patients with *BRAF*^V600E^ metastatic melanoma, treatment of Vemurafenib, Dabrafenib, and Encorafenib resulted in dose-dependent tumor suppression and improved overall survival rates relative to other therapeutic approaches.^324,325^ Notably, patients with *BRAF*-mutant melanoma have displayed significant responses to BRAF inhibitors such as vemurafenib (PLX4032, RG7204) and Dabrafenib (GSK2118436), while those with *BRAF* wild-type melanoma have not shown similar responses.^326^

Despite the encouraging beneficial observed with RAF inhibitors, approximately half of the patients treated with RAFi experience cancer recurrence due to the development of resistance to the RAF inhibitors within 6–7 months of initiating treatment.^327^

##### MEK inhibitors (MEKi)

While activated *MEK* mutations are relatively rare in human tumors, mutations in upstream genes like *RAS* or *RAF* are implicated in over 85% of malignancies, often resulting in elevated MEK activity.^328^ Targeting MEK has thus emerged as a promising and cutting-edge therapeutic approach. Combinations of MEK and BRAF inhibitors have received FDA approval and have proven effective in inhibiting tumor growth in both preclinical models and patients with RAS or RAF mutations.^329^ One notable MEK inhibitor, Trametinib, was identified progression-free and overall survival in patients with *BRAF*^*V600E*^-mutated metastatic melanoma.^330^ Furthermore, combinations of Dabrafenib and Trametinib led to improved progression-free survival in patients with metastatic *BRAF*^V600E^-mutated melanoma.^331^ Cobimetinib (GDC-0973) is another highly selective allosteric MEKi that has demonstrated effectiveness in *BRAF*^V600E/K^-mutant patients as well as in cell lines with BRAF*-* or KRAS-mutations.^332^ The FDA approved Cobimetinib in combination with Vemurafenib for the treatment of *BRAF*^V600E/K^*-*mutant and unresectable melanoma in 2015.^333^ Binimetinib, also known as MEK162, ARRY-162, or ARRY-438162, is an effective oral MEKi approved for patients with *BRAF*^*V600E/K*^*-*mutant and unresectable melanoma. The FDA approved the combination therapy of Binimetinib and Encorafenib in 2018^334^. In 2020, Selumetinib (KOSELUGO, AstraZeneca), a highly specific MEKi, is approved for the treatment of pediatric children with neurofibromatosis type 1 (NP1) who have symptomatic, inoperable plexiform neurofibromas (PN).^335^ Selumetinib is also under evaluation for use in other cancers such as melanoma, gliomas, and non-small cell lung cancers, where MEK1/2 is overexpressed.^336^

#### Ongoing clinical studies on the combination of BRAFi and MEKi

In patients with *BRAF* mutated cancer, a molecular mechanism analysis has revealed potential target for RAS/RAF/MAPK inhibitors. This analysis has led to a significant shift in the treatment approach for patients with *BRAF* mutated cancer. It is believed that inhibiting both BRAF and MEK greatly can improve clinical outcomes for melanoma patients by slowing or preventing the development of resistance to BRAFi alone.^337^ In Table 3, we have compiled an update overview of clinical studies related to combination of BRAFi and MEKi based on information from the ClinicalTrials.go database.

**Table 3:** Combinations of BRAFi and MEKi under clinical investigations

Targeted therapy	Condition (s)	Phase	Participants	Estimated study completion	Current status/Other ID	NTC identifier
Vemurafenib + cobimetinib	BRAF^V600E^, PCP	II	36	August 2026	Recruiting/NCI-2017-00740	NCT03224767
	HM, melanoma, TC, PON, CRN, NSCLC, Gloma, MM, and ATC	II, III	30	September, 2029	Recruiting/CRUKD/21/004	NCT05768178
	Melanoma	II	24	January 2024	Active, not recruiting/ML28606	NCT02036086
Encorafenib + binimetinib	Advanced BRAF-mutant cancers	I, II	17	September, 2023	Completed, no result posted/18-547	NCT03843775
	Solid tumor	II	26	December, 2023	Recruiting/BEAVER-001	NCT03839342
	Locally advanced, metastatic, recurrent and stage III/IV PC AJCC v8	II	29	December, 2024	Active, not recruiting/NCI-2020-02972	NCT04390243
	NSCLC	II	55	March, 2025	Recruiting/CTR20212962	NCT05195632
	HCL	II	45	July, 2024	Recruiting/200076	NCT04324112
	NSCLC	II	98	June, 2024	Active, not recruiting/ARRAY-818-202	NCT03915951
	Melanoma stage III, in-transit metastasis of CM	II	28	January, 2026	NL77905.058.21	NCT05767879
	Relapsed or refractory MM with BRAF^V600E/K^ mutation	II	12	December, 2022	Active, not recruiting/CLGX818ADE01T	NCT02834364
Dabrafenib + trametinib	Melanoma, NSCL, solid tumor, rare cancers, and HGG	IV	100	December, 2027	Recruiting/CDRB436X2X02B	NCT03340506
	Stage IIIB/C CM AJCC v7	II	66	April, 2024	Active, not recruiting/NCI-2014-01969	NCT02231775

*PCP* papillary craniopharyngioma, *HM* hematologic malignancy, *TC* thyroid cancer, *PON* papillary ovarian neoplasms, *CRN* colorectal neoplasms, *LNC* laryngeal neoplasms carcinoma, *NSCLC* non-small-cell lung, *MM* multiple myeloma, *ECD* erdheim-chester disease, *ATC* anaplastic thyroid carcinoma, *PC* pancreatic carcinoma, *HCL* hairy cell leukemia

##### Vemurafenib and Cobimetinib

Hoffmann-La Roche/Genentech sponsored a Phase III clinical trial that employed a double-blind, placebo-controlled design to compare Vemurafenib alone with a combination of Vemurafenib and Cobimetinib (GDC-0973). This trial focused on patients who had not received prior treatment and were diagnosed with *BRAF*^V600^*-*mutattion-positive, unresectable locally advanced, or metastatic melanoma (*NCT01689519*). Although there was a slight increase in treatment-related side effects, the addition of Cobimetinib to Vemurafenib led to a significant improvement in progression-free survival (PSR) among patients with *BRAF*^*V600*^*-*mutated metastatic melanoma.^337^ Also, a recent clinical study demonstrated that the combination of Vemurafenib and Cobimetinib produced a partial response or better in 15 out of patients with papillary craniopharyngiomas, as part of a single-group study (*NCT03224767*). The average reduction in tumor size was 91%, and progression-free survival (PFS) was 87% at 12 months (95% CI, 57 to 98) and 58% at 24 months.^338^ Numerous ongoing studies are currently underway, with participants either receiving intervention or undergoing examination to assess the clinical and pathological responses to Vemurafenib and Cobimetinib in *BRAF*-positive cancers (*NCT05768178, NCT02036086*).

##### Encorafenib and Binimetinib

An observational study has been initiated to investigate the real-world effectiveness, impact on quality of life, safety, and tolerability of Encorafenib in combination with Binimetinib for the treatment of unresectable advanced or metastatic BRAF cancers in Germany, Austria and Switzerland (*NCT04045691*). Several clinical trials are currently underway, with participants either receiving an intervention or undergoing examination to assess the safety, tolerability and efficacy of combining Encorafenib with Binimetinib in patients with *BRAF*^V600^-mutant metastatic cancers, including pancreatic cancer (PC) (*NCT04390243*), NSCLC (*NCT05195632*, *NCT03915951*), hairy cell leukemia (HCL) (*NCT04324112*), multiple myeloma (MM) (*NCT02834364*) and melanoma (*NCT05767879*) Despite the significant improvement in survival seen with combination therapy involving of BRAFi and MEKi for *BRAF*^V600^-mutant melanomas, there remain limited options for targeted therapy in cases of BRAF^nonV600^-mutant melanomas. It has been observed that *BRAF*^V600^ mutations often co-occur with NF1 deletion, and that these mutations can signal as monomer and dimers when NF1 loss present.^339^ Consequently, in *BRAF*^nonV600^-mutant melanomas with co-occurring NF1 loss-of-function mutations, the combination of BRAFi targeting both monomeric and dimeric BRAF, along with MEK inhibition, has been shown to significantly reduce cell viability in vitro and tumor growth in vivo. Recently, two ongoing clinical studies are exploring whether patients with *BRAF*^*nonV600*^-mutant melanomas may benefit from the currently FDA-approved combination therapy of Encorafenib (BRAFi) and Bonimetinib (MEKi), which is reserved for *BRAF*^*nonV600*^-patients (*NCT03839342*, *NCT03843775*). These results are considered hypothesis-generating and will need further confirmation in larger clinical trials in the future.

##### Dabrafenib and Trametinib

Recent reports have shown that previously untreated individuals with metastatic melanoma and *BRAF*^*V600E/K*^ mutations exhibited superior responses to the combination therapy of Dabrafenib and Trametinib compared to Dabrafenib alone.^340^ The randomized Phase III study funded by GlaxoSmithKline compared the efficacy of combination therapy involving Dabrafenib and Trametinib to that of administered alongside a placebo (Dabrafenib monotherapy) (*NCT01584648*). Moreover, the Novartis designed non-interventional study has initiated to evaluate the use of Dabrafenib in combination with Trametinib as adjuvant treatment for Chinese patients with stage III melanoma carrying the *BRAF*^*V600E*^ mutation after complete resection (*NCT04666272*). Additionally, a managed access program (MAP) cohort clinical trial provides guidelines to physician for the treatment and monitoring of Trametinib/Dabrafenib, in eligible patients diagnosed with advanced NSCLC featuring the *BRAF*^*V600E/K*^*-*activating mutations (*CTMT212X2002I*, *NCT04507919*).

#### Assessment of a dual RAF/MEK inhibitor: VS-6766

A novel RAF/MEK inhibitor CH5126766/RO5126766 was discovered while screening for compounds that induce p27. It exhibits more prolonged inhibition of MAPK signaling compared to PD0325901, a well-known ATP-competitive MEK inhibitor.^341^ VS-6766, also known as CH5126766, is a first-in-class dual RAF-MEK inhibitor currently under investigation as a potential treatment for multiple myeloma and solid tumors harboring different *RAS-RAF* mutations. It targets two key nodes in the RAS/RAF/MAPK signaling pathway, demonstrating both safety and promising efficacy.^342^ Numerous interventional clinical trials are ongoing to evaluate the effectiveness of VS-6766, both as a standalone therapy and in combination regimens (Table 4). Notably, an intermittent dosage regimen of VS-6766 and Defactinib has shown clinical activity in patients with recurrent low-grade serous ovarian cancer (LGSOC). These findings have paved the way for an ongoing registration-directed study investigating combination of VS-6766 and Defactinib in patients with recurrent LGSOC (*NCT04625270*).

**Table 4:** Ongoing interventional clinical trials of VS-6766 (Dual RAF/MEK inhibitor) for cancer therapy

NTC identifier	Adjunctive agent (s)	Conditions	Phase/Participants	Study status/Estimated completion date
NCT00773526	-	Neoplasms	Phase I/18	Completed/Sep 2011
NCT05187169	-	Food effect	Phase I/52	Completed/April 2022
NCT03875820	Defactinib	NSCLC, LGSOC, EEC, PC	Phase I/87	Active, not recruiting/Oct 2023
NCT05512208	Defactinib	EEC, MOC, HGSOC, CC	Phase II/55	Recruiting/Dec 2029
NCT05787561	Defactinib	MGC	Phase II/20	Recruiting/March 2025
NCT04625270	Defactinib	OC, LGSOC, adenocarcinoma	Phase II/225	Recruiting/Dec 2026
NCT04620330	Defactinib	NSCLC, KRAS mutation	Phase 2/100	Recruiting/Dec 2025
NCT02407509	Everolimus	Solid tumors, MM, LC, OC	Phase II/104	Recruiting/May 2024
NCT04720417	Defactinib	Metastatic UV	Phase II/18	Recruiting/Sep 2023
NCT05798507	Defactinib	GBM	Early phase I/12	Recruiting/Sep 2024
NCT06007924	Defactinib	TC	Phase II/30	Recruiting/Aug 2027
NCT05669482	Defactinib, gemcitabine, nab-paclitaxel	KRAS mutation, PDAC	Phase I, II/40	Recruiting/May 2025
NCT05375994	Adagrasib	NSCLC, KRAS mutation, LC, MND	Phase I, II/58	Recruiting/July 2024
NCT05608252	Abemaciclib, fulvestrant	Met HR + /HER- BC	Phase I, II/63	Recruiting/Dec 2027
NCT05200442	Cetuximab, Pill Diary	CRC, COADREAD, mCRC	Phase I, II/53	Recruiting/April 2024
NCT05074810	Sotorasib	NSCLC, KRAS mutation	Phase I, II/53	Recruiting/Dec 2023

*NSCLC* non-small cell lung cancer, *LGSOC* low-grade serous ovarian cancer, *PC* pancreatic cancer, *EEC* endometrioid cancer, *MOC* mucinous ovarian cancer, *HGSOC* high-grade serous ovarian cancer, *CC* cervical cancer, *MM* multiple myeloma, *LC* lung cancer, *OC* ovarian cancer, *MND* malignant neoplastic disease, *MGC* mesonephric gynecologic cancer, *BrC* Breast cancer, *CRC* colorectal cancer, *mCRC* metastatic colorectal cancer, *COADREAD* colorectal adenocarcinoma, *LC* lung cancer, *Met HR*^*+*^*/HER*^*−*^ *BC* metastatic hormone receptor-positive breast cancer, *PDAC* pancreatic ductal adenocarcinoma, *mUV* metastatic uveal melanoma, *GBM* glioblastoma, *TC* thyroid cancer

#### Targeted therapy in pediatric BRAF-mutational gliomas

The *BRAF*^*V600E*^ mutations gas been associated with reduced responsiveness to conventional treatment in pediatric low-grade gliomas (pLGG).^343^ Recent findings from the phase II TADPOLE trial suggest that targeted therapy focusing on the RAS/RAF/MAPK signaling is beneficial for pediatric brain tumors with *BRAF* mutations. In earlier clinical studies, Dabrafenib demonstrated significant clinical efficacy and tolerability, both as monotherapy and in combination with trametinib, for the treatment of pLGG with *BRAF*^V600E^ mutations (*NCT01677741*). These results have spurred further exploration of this combination as a first-line therapy (*NCT02684058*). Notably, Dabrafenib plus Trametinib, when used as a first-line therapy, achieved significantly higher response rates, longer progression-free survival, and improved safety profiles compared to standard monotherapy in pLGG with *BRAF*^*V600E*^ mutations.^344^ In this randomized study, the combination therapy of Dabrafenib and Trametinib showed an overall response rate (ORR) of 47%, while monotherapy yielded an ORR of only 11%. Furthermore, the Dabrafenib and Trametinib combination significantly extended median progression-free survival compared to chemotherapy (20.1 months vs. 7.4 months) and led to clinical improvement in 86% of patients, as opposed to 46% in the monotherapy group. Additionally, the study with *BRAF*^*V600E*^ -mutant pediatric high-grade gliomas (pHGG) demonstrated that combining Dabrafenib with Trametinib exhibits an enhanced ORR of 56 %, associated with long-lasting responses and improved duration of response (22.2 months), overall survival (32.8 months), and along with a favorable safety profile, in relapsed/refractory *BRAF*^*V600*^-mutant pHGG patients. In 2023, the FDA granted its approval for the utilization of the Dabrafenib and Trametinib combination as the primary treatment for pLGG in children aged 1 year or older. This milestone marks the successful outcomes of the TADPOLE trials in the pLGG population. Moreover, in 2022, the FDA also sanctioned the use of this combination therapy for patients aged six and above who were dealing with relapsed/refractory *BRAF*^*V600*^ solid tumors. This additional approval serves to reinforce the positive results observed in the pHGG cohort.

#### Combining BRAF and MEK inhibitors with other targeted therapy

Combining BRAF and MEK inhibitors with other targeted therapies is currently under investigation, aiming to prolong tumor responses.^345^ These therapies are also gaining traction in neoadjuvant and adjuvant settings (see Table 5).

**Table 5:** Combinations of other targeted therapy along with RAF/MAPK inhibitors under clinical investigations

Targeted therapy	Condition (s)	Phase	Participants	Estimated study completion	Current status/Other ID	NTC identifier
PF-07284890, binimetinib, midazolam	Malignant melanoma carcinoma, NSCLC, brain neoplasms, malignant neoplasms	I	57	November 2023	Active, not recruiting/C4471001	NCT04543188
PF-07799933, binimetinib, cetuximab	Melanoma, CRC, NSCLC, TC, Glioma	I	174	January 2028	Recruiting/C4761001	NCT05355701
Encorafenib, PF07799544, PF07284890, PF07799933,	Melanoma, glioma, TC, NSCLC, malignant neoplasms, brain neoplasms, CRC	I	124	March 2027	Recruiting/C4901001	NCT05538130
Spartalizumab, dabrafenib trametinib	Melanoma	III	568	December 2023	Active, not recruiting/CPDR001F2301	NCT02967692
Encorafenib, nivolumab, ipilimumab, binimetinib	Melanoma	I, II	2	August 2023	Active, not recruiting/HCC 20-190	NCT04655157
Dabrafenib, trametinib pembrolizumab	Melanoma	II	60	November 2024	Active, not recruiting/MIA2015/179	NCT02858921
Dabrafenib, trametinib, Pembrolizumab	TGAC, TGSCC	II	30	June 2024	Recruiting/NCI-2020-09803	NCT04675710
Encorafenib, Binimetinib, Nivolumab	Melanoma, skin cancer	I	13	May 2028	Active, not recruiting/MCC-19441	NCT03543969
Dabrafenib trametinib, nilotinib	Metastatic melanoma, BRAF mutation	I	15	March 2027	Recruiting/MCC-20-MEL-11-PMC	NCT04903119
Vemurafenib, HL-085	Solid tumor	I	45	December 2023	Recruiting/HL-085-102	NCT03781219
ABM-1310, cobimetinib	Advanced solid tumor, BRAF^V600^ mutation	I	112	January 2025	Recruiting/ ABM1310X1101	NCT04190628
Mirdametinib, BGB-3245	Advanced solid tumor	I, II	136	June 2027	Recruiting/MEKRAF-AST-101	NCT05580770
XL888, Vemurafenib, Cobimetinib	Melanoma, skin cancer	I	26	October 2023	Active, not recruiting/MCC-18597	NCT02721459
AT13387, Dabrafenib, trametinib	Clinical and pathological stage III, IIIa/b/c/d CM AJCC v6/7	I	22	March 2019-	Active, not recruiting/NCI-2014-00615	NCT02097225
Trametinib, CDK4/6	Melanoma	II	1000	December 2028	Recruiting/MIA2015/174	NCT02645149
Vemurafenib, tiragolumab atezolizumab, cobimetinib,	Clinical and pathological stage III, IIIa/b/c/d CM AJCC v8	I	30	June 2024	Recruiting NCI-2018-01018	NCT03554083
Dabrafenib, trametinib, PDR001	mCRC	II	25	December 2022	Recruiting/18-144	NCT03668431
Dabrafenib, Trametinib, HCQ	Brain LGG and HGG with BRAF aberration and brain LGG with NP1 aberration	I, II	75	June 2027	Recruiting/PBTC-055	NCT04201457
Dabrafenib, tazemetostat, trametinib	Clinical stage IV CM AJCC v8,Metastatic melanoma	I, II	58	December 2024	Recruiting/NCI-2020-07044	NCT04557956
Dabrafenib, trametinib, radiation	AA, AGG, APXA, GBM, malignant grade 3 glioma	II	58	September 2027	Recruiting/NCI-2019-02289	NCT03919071

*CM* cutaneous melanoma, *AJCC v8* American joint committee on cancer version 8, *mCRC* metastatic colorectal cancer, *LGG* low grade glioma, *HGG* high grade glioma, *NP1* neurofibromatosis type 1, *AA* anaplastic astrocytoma, *AGG* anaplastic ganglioglioma, *GBM* glioblastoma, *HCQ* hydroxychloroquine, *mCRC,* metastatic colorectal cancer, *APXA* anaplastic pleomorphic xanthoastrocytoma, *CRC* colorectal cancer, *NSCLC* non-small cancer lung cell, *TC* thyroid cancer, *TGAC* thyroid gland anaplastic carcinoma, *TGSCC* thyroid gland squamous cell carcinoma

Patients with *BRAF*^*V600*^-mutant malignancies are benefiting from RAF/MEKi inhibitor therapy. However, long-term efficacy is limited by disease progression in the brain due to inadequate pharmacokinetics (PK) and pharmacodynamics (PD).^346,347^ Researchers are working on enhancing the intrinsic properties of drugs, such as their PK and PD, to increase the effectiveness of RAS/RAF/MAPK inhibitors. One promising molecule is PF-07284890 (BRAFi), a potent brain-penetrant compound, currently undergoing evaluation in a first-in-human trial for patients (*NCT05538130*, *NCT04543188*).

Similar to BRAF/MEKi, combining adjuvant therapy together with specific checkpoint inhibitors, such as anti-PD-1 (Nivolumab and Pembrolizumab) or anti-CTLA-4 (Ipilimumab), has shown promising results. It leads to extended recurrence-free survival and a reduced occurrence of severe adverse events in patients who have undergone resection for stage IIIB, IIIC, or IV melanoma (*NCT02388906, NCT04949113, NCT01972347*). Additionally, the combination of these immune checkpoint inhibitors with BRAF/MEK-targeted medications appears to be a more viable treatment option due to their immunomodulatory action (*NCT02967692, NCT04655157, NCT02858921*). Furthermore, a phase I trial study has demonstrated the potential synergy between anti-PD-1 inhibitor and other BRAF/MEK inhibitors in treating patients with *BRAF*-mutant stage IIIC-IV melanoma (*NCT03543969*). Meanwhile, a phase II trial is evaluating the impact of Pembrolizumab, Dabrafenib, and Trametinib as a neoadjuvant therapy for patients with *BRAF*^*V600E*^-mutated anaplastic thyroid cancer (ATC) before surgical intervention (*NCT04675710*). An ongoing neoadjuvant phase II study of Dabrafenib, Trametinib and/or Pembrolizumab in *BRAF*^*V600*^-mutant respectable stage IIIB/C melanoma is investigating the effect of Pembrolizumab, Dabrafenib, and/or Trametinib in reducing tumor sizes before surgery and preventing melanoma recurrence after surgery in patients with *BRAF*^*V600*^-mutant respectable stage IIIB/C melanoma (*NCT02858921*).

However, a randomized phase II trial, advanced melanoma patients with *BRAF*^V600E/K^ mutations who received a combination of Dabrafenib (BRAFi), Trametinib (MEKi), and Pembrolizumab (a PD-1-blocking antibody) displayed numerically longer progression-free survival (PFR) and duration of response but experienced with a higher rate of grade 3/4 adverse events compared to those treated with the doublet therapy of Dabrafenib and Trametinib along with a placebo (*NCT02130466*).

In addition to the established interventions using BRAF and MEK inhibitors, HSP90 inhibitors for patients with the *BRAF*^*V600*^-mutant melanoma are under investigation due to their manageable side-effect profiles when combined with BRAFi.^348^ HSP90 inhibitors, such as AT13387 or XL888, offer a promising strategy to combat drug resistance resulting from BRAF and MEK inhibition in melanomas.^348^ Clinical trials investigating the combination of BRAF/MEKi with XL888 or AT13387 are currently underway (*NCT02721459, NCT02097225*). Moreover, targeting the CDK4 pathway has emerged as a therapeutic approach for patients with *BRAF*-mutant metastatic melanomas.^349^ A patient-centered translational study has shown improved clinical outcomes for individuals with stage III or stage IV metastatic *BRAF* and *NRAS* wild-type melanoma who do not respond to standard therapy, typically immunotherapy. Lastly, there are ongoing clinical trials exploring various combinations with BRAF/MEKi, which information can be found in Table 5, provided by ClinicalTrials.gov.

### RAFi resistance

The discovery of small-molecule BRAF inhibitors was the start of a revolution in the treatment of advanced melanoma.^324,350^ Some BRAF inhibitors have demonstrated promising results for the treatment of patients with melanoma featuring the BRAF^V600E^ mutation. Despite the exceedingly positive effects of these inhibitors, the development of drug resistance is a widely occurring phenomenon in patients with cancer.^16,351^ RAFi resistance can be characterized as either intrinsic acquired or adaptive.^14^

#### Intrinsic RAFi resistance

Although cancers featuring activating BRAF mutations respond well to RAFi, a considerable fraction of melanoma cells harboring the *BRAF* ^*V600E*^ mutation show inherent resistance to RAFi.^8^ *BRAF*^V600E^–mutant tumors do not sensitize to either Vemurafenib or Trametinib due to high cyclin D1 and YAP1 expression.^8,352–354^ Activation of phosphatase and tensin homolog (PTEN), forkhead box O3, AKT, or insulin-like growth factor 1 (IGF-1) generally leads to the development of intrinsic RAFi resistance due to the suppression of apoptosis mediated by BCL2-interacting mediator of cell death.^355–358^ Intrinsic RAFi resistance represents an adaptation mediated by transcriptional and epigenetic rewiring.^359^ Relief from the human EGFR 3 feedback inhibition mediated by RAFis and MEK inhibitors reduces the anticancer effects of these treatments against *BRAF*-mutant thyroid carcinomas.^360^ Human growth factor (HGF) released by stromal cells activates the HGF receptor (MET), reactivating the MAPK and PI3K–AKT signaling pathways, resulting in the rapid development of RAFi resistance.^361^

#### Acquired RAFi resistance

Acquired RAFi resistance generally develops due to the competitive burden introduced by drug treatment, which results in diverse genetic abnormalities or the acquisition of therapy-induced de novo cellular epigenetic and transcriptional reprogramming events.^362^ In nearly all cases, acquired drug resistance is linked to secondary mutations in *RAF*, leading to the activation of parallel signaling pathways (such as RTKs) and the “on-target” accumulation of mutations in the RAF pathway.^363–368^ These secondary mutations often occur at “gatekeeper” residues within a RAF ATP-binding site, preventing inhibitors from binding to the existing hydrophobic pocket of the ATP-binding domain. A BRAF mutation at Thr259 confers resistance to some anticancer reagents (e.g., SB590885 and PLX4720), although intrinsic kinase activity is maintained.^363^ Acquired resistance to Vemurafenib is related to the increased expression of several RTKs, especially PDGF receptor. In addition, CRAF overexpression leads to acquired resistance to BRAF inhibition mediated by a RAF kinase switch in melanoma.^368,369^ MAP3K8 (COT) reactivates ERK in some melanoma samples that are resistant to RAFis and MEK inhibitors.^370,371^

#### Adaptive RAFi resistance

Adaptive resistance to RAFi arises from internal compensating mechanisms, often termed adaptive responses or feedback loops. These mechanisms enhance the survival and proliferation potential of a subset of the initial tumor population, ultimately promoting tumor growth. Such adaptive RAFi resistances can manifest in two main forms: non-transcriptional adaptive response, which regulate post-translational modifications of upstream kinases, and transcriptionally mediated adaptive responses. Many of these inherent resistance mechanisms are primarily underscored by the reactivation of the MAPK signaling pathway, consequently, reduces patient responses. Notably, the reactivation of MAPK via paradoxical signaling (as described in section 4.3) accounts for a significant portion of adaptive mechanisms, contributing to the diminished sensitivity to pathway-targeted drugs and overall patient responses.^372^

### RAFi adaptation to RAF dimerization: feedback inhibition and paradoxical activation in RAF-mutant cancers

**Fig. 8:**
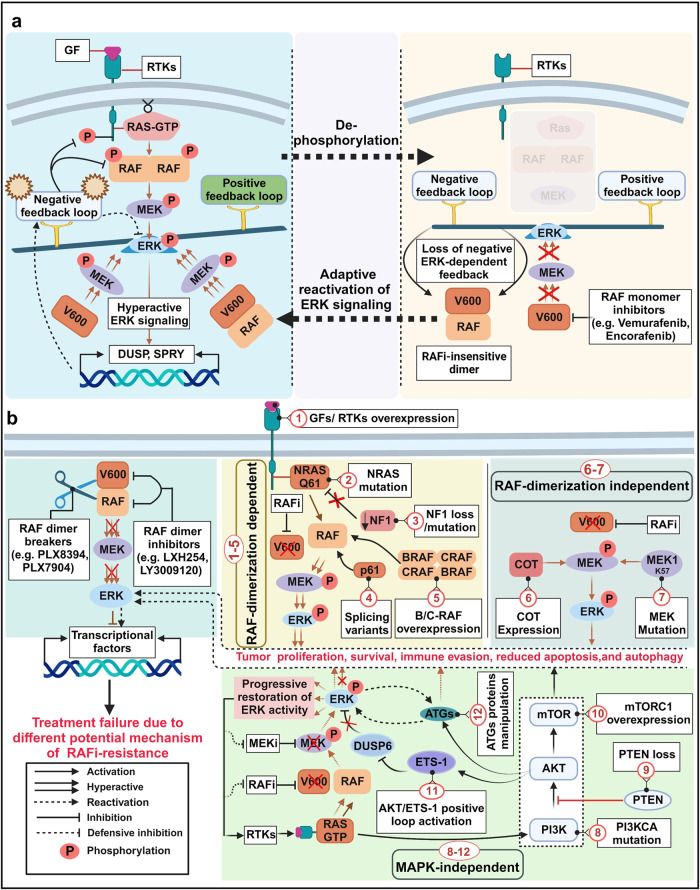
Mechanistic basis of RAF inhibitor (RAFi) resistance. **a** RAFi resistance in *RAF*-mutant cancer. BRAF^V600E^–mutant cancers are characterized by hyperactive extracellular signal-related kinase (ERK) signaling during early disease, resulting in negative feedback inhibition of upstream signaling. RAS-GTP expression is minimal, but monomeric BRAF^V600E^ responds to signals. Under these conditions, RAFi inhibits ERK signaling. Subsequent treatment with Vemurafenib suppresses ERK-dependent negative feedback, restoring receptor tyrosine kinase (RTK) signaling. Despite the presence of RAFi, RTK signal restoration elevates RAS-GTP levels, drives the formation of RAFi-insensitive RAF dimers, and reactivates ERK signaling. Over time, negative feedback pathways are partially restored, and a new steady-state condition featuring reactivated ERK signaling develops. **b** RAFi resistance mechanisms. RAFi resistance promotes significant RAF dimerization through growth factors or RTK activation, NRAS mutation (NRAS Q61), NF1 loss, expression of RAF splice variants (p61), or overexpression of BRAF or CRAF. Reactivation of ERK signaling and RAFi resistance can also develop in a RAF dimerization–independent manner involving MEK mutations or RAF bypass activation by COT (an ERK kinase kinase). This figure was created with BioRender.com

Aberrant ERK1/2 signaling can lead to oncogenicity, depending on the extent of ERK1/2 activation^373^. ERK dysregulation is associated with cell death and senescence,^374,375^ and the feedback mechanisms in ERK signaling are closely connected to cellular dysfunction.^376^ The negative feedback of ERK signaling is regulated directly via ERK pathway phosphorylation^376^ or indirectly through de novo gene expression, such as those encoding dual-specificity phosphatases (DUSPs) and Sprouty (SPRY).^377,378^ ERK-mediated feedback regulation directly targets RAF proteins, reducing dimerization.^379^ RAFi resistance reactivates ERK signaling and induces RAFi insensitivity via mechanisms that bypass RAF-dependent signaling.^322^ ERK reactivation is very subtlety regulated at multiple levels via “intra-pathway” feedback loops and mediated by phosphorylation, intracellular localization, and complex formation (Fig. 8a).^380^. Under normal conditions, RAS-activated RAF dimerizes and phosphorylates the downstream effectors MEK and ERK. Interestingly, ERK also activates other genes (e.g., *DUSPs* and *SPRY*) that inhibit the ERK pathway via a negative feedback loop, resulting in RAS dephosphorylation, which weakens the signal.^14,15,378,381–383^ The network of negative feedback loops limits ERK activation driven by the direct phosphorylation of components in the MAPK cascade.^384–386^ In *BRAF*-mutant cancers, the MEK–ERK axis is constitutively active, independent of RAS-mediated RAF dimerization, and low levels of basal RAS activation combined with direct phosphorylation result in the strong activation of negative feedback regulators (SPRY and DUSP), inhibiting signaling intermediates, such as EGFR and SOS. As a result, signaling is hampered downstream of activated receptors in *BRAF*^V600E^–mutant cells. Treatment with Vemurafenib, a first‐generation ATP-competitive RAFi, results in a temporary reduction in ERK activity,^324^ which rapidly recovers in *BRAF*-mutant cancer cells,^387^ possibly due to the inhibition of ERK-dependent negative feedback.^15,388^ The restoration of RTK signaling increases RAS-GTP levels, followed by an increase in the homo- or heterodimerization of RAF, which paradoxically reactivates ERK signaling in the presence of RAFis.^389^ This paradoxical activation of RAFi-insensitive *BRAF*^*V600E*^–mutant dimers result in the formation of a new steady-state signaling network, even in the presence of RAFis.^389,390^ Thus, RAFi are ineffective against these tumors due to the development of constitutive BRAF-mutant dimers or the transactivation of BRAF–CRAF heterodimers.^389,391^

New RAFi families, such as pan-RAF dimer inhibitors and paradox breakers (dimer breakers), appear to be more effective therapeutic options than previous monomeric-targeting RAFis in tumors with RAF dimer–induced signals (Fig. 8b).^392–394^ pan-RAF dimer inhibitors (e.g., LXH254 and LY3009120) bind directly with RAF dimeric domains and inhibit RAF dimerization in *BRAF*^V600E^–mutant tumors. However, RAS mutation or amplification, *BRAF*^V600E^ amplification, and intragenic deletion or *BRAF* splice variants can lead to RAFi resistance in patients with *BRAF*^V600E^–mutant tumors.^390,395,396^ Importantly, these inhibitors target both active RAF dimers and monomers to block ERK signaling in cancer cells.^397^ Most pan-RAF inhibitors (e.g., AZ628, Belvarafenib, CCT196969, CCT241161, LY3009120, and TAK-580 [MLN2480]) are successful when tested using in vitro cellular assays.^398,399^ However, the use of these inhibitors in patients has been limited by a lack of selectivity for mutant BRAF.^400^ Although LY3009120 exhibits antitumor effects in patients with mutant RAF dimers, persistent treatment with this drug causes severe resistance due to *BRAF*^*V600E*^ dimerization.^390^ A limited dose escalation has been examined to determine the maximally tolerated dose in patients with *RAS*-mutant cancer, indicating the potential of this drug as an anticancer therapy.^400^ LXH254, an inhibitor of RAF dimerization, shows specific paralog selectivity, preventing dimeric formation of BRAF and CRAF but not ARAF.^397^ These drugs are unable to suppress MAPK signaling in normal cells because they inhibit mutant RAF dimers and monomers at great potency compared with their effects on wild-type RAF dimers. As a result, V600E–mutant BRAF that dimerizes with other RAF mutants or with wild-type BRAF or CRAF exhibit drug resistance due to the unintended paradoxical activation of ERK,^322,389,393^ which may also be responsible for the elevated toxicity and disruption in RAF signaling observed in non-cancer cells. As a result, these medications prove ineffectual when confronted with tumors carrying BRAF^nonV600E^-mutations. Additionally, an increase in the expression of BRAF^V600E^ -dimers can lead to acquire resistance, primarily because of unintentional paradoxical activation of ERK.^390^ This paradoxical activation might also be accountable for heightened toxicity and disturbances in RAF signaling, which are observable in non-cancer cells.^401^

In addition to pan-RAF dimer inhibitors, paradox breakers (e.g., PLX8394 and PLX7904) disrupt homo- and heterodimers that contain BRAF, resulting in reduced paradoxical ERK activation in cells expressing both mutant and wild-type RAF.^394,402^ These inhibitors selectively bind BRAF to suppress ERK1/2 in *BRAF*^V600E^–mutant cells without inducing the paradoxical activation of ERK1/2 observed in *RAS*-mutant cells. PLX8394 specifically inhibits BRAF in colon adenocarcinoma cells, preventing the paradoxical activation of the MAPK pathway^403^ with greater potency than Encorafenib, a selective inhibitor of active BRAF monomers, in *BRAF*-mutant colorectal cancer.^404^ However, PLX8394 deteriorates ERK signaling via the specific dissociation of BRAF–containing dimers (BRAF homodimers and BRAF–CRAF heterodimers) but has no effect on CRAF homodimers or dimers containing ARAF due to differences in the amino acid residues within the N-terminal region of the kinase domain among RAF isoforms.^402^ In addition, PLX8394 activates RAF signaling via BRAF dimerization in several melanoma cells.^405^ RTK-induced mammalian target of rapamycin (mTOR) activity and other bypass signaling may be able to compensate for decreased p-ERK levels when using paradox breakers.^406^

The ability of *RAF*-mutant tumors to adapt to RAFi is primarily due to an increase in RAF dimerization (Fig. 8b, *RAF dimerization–dependent*).^106^ Mutant RAF dimerization is often associated with RAFi resistance. An inhibitory bypass mechanism can also result in resistance to RAFi, via RAF-independent ERK reactivation (Fig. 8b, *RAF dimerization–independent*). For example, mutations in the MEK kinase COT (MAP3K8) and MEK1 (encoded by MAP2K1) are closely related to inhibitory bypass resistance that develops in response to RAFi.^371,407^ RAFi-resistant tumor cells show the rapid recovery of MAPK pathway activation, allowing escape from RAFi therapy; therefore, the total blockade of the entire pathway is essential for stimulating apoptosis in *RAF*-mutant cancers.^408^ The combination of RAFi together with other downstream inhibitors (e.g., MEK inhibitors) can maximize MAPK pathway inhibition and minimize cancer resistance. Compared with typical single-agent chemotherapy, Trametinib, a MEK inhibitor, enhances progression-free and overall survival rates in patients with metastatic melanoma who harbor a *BRAF*^*V600E/V600K*^ mutations.^19^ However, *BRAF*^*V600E*^–mutant tumors still can develop resistance to combination RAFi and MEK inhibitor therapy over the course of months to years.^409^ Most patients who are treated with RAFi and MEK inhibitor combination therapy develop mild (Grade 1–2) toxicity in response to these drugs.^22,410,411^ Therefore, a solution to the underlying toxicity induced by RAFi is necessary to develop effective therapies for *RAF*-mutant malignancies.

## Strategies to overcome chemoresistance related to targeting the RAS/RAF/MAPK pathway

The hyperactivation of the RAS/RAF/MAPK signaling pathway plays a significant role in the development of numerous human malignancies. While current targeted therapies like BRAFi and MEKi show varying levels of effectiveness across different cancer types, s substantial number of patients develop resistance relatively quickly.^412^ Ongoing cancer research is now actively addressing the challenge of countering both intrinsic and acquired drug resistance to small-molecule RAF inhibitors. Strategies involving combination therapies are been exploring as potential approaches to address RAF inhibitor resistance.^413^

### Utilizing existing combination therapeutic strategies against RAFi resistance

The RAS/RAF/MAPK inhibitors currently in use have been through clinical development as combination therapies. They can either vertically target specific proteins within the MAPK pathway or horizontally inhibit multiple signaling pathways.^414^

#### Vertical pathway inhibition

*BRAF*-targeted single-agent therapy frequently results in the development of drug resistance due to MAPK pathway reactivation. The vertical blockade of MAPK signaling mediated by RAFi may be supplemented by other agents targeting the intermittent pathway (RTK–RAF–MEK–ERK).^415–417^ Combination therapy has become an important therapeutic goal in class-specific polytherapy.^417,418^ A vertical blockade combining the RAFi Dabrafenib with the EGFR inhibitor Panitumumab and the MEK inhibitor Trametinib is able to greatly suppress MAPK signaling, demonstrating improved therapeutic efficacy in metastatic colon cancer compared with other therapies.^419,420^ Although several therapeutic approaches using various medication doses and regimens can extend therapeutic responses, these strategies can also accelerate the development of resistance mechanisms. Continuous drug-related toxicity is also a risk, and the intermittent targeting of mutant *BRAF* can lead to the development of new drug-resistant tumors.^418,421–423^ As Vemurafenib-resistant cancer cells are reliant on the presence of the drug, withdrawal of drug treatment could be sufficient to induce tumor regression.^418,422^ Although the development of resistance to multiple inhibitors targeting the MAPK pathway remains to be addressed, this drug combination approach improves patient outcomes.^424,425^

#### Horizontal pathway inhibition

Targeting multiple signaling pathways simultaneously, known as horizontal pathway inhibition, can be an effective strategy to counteract potential compensatory or escape mechanisms that selective pathway inhibition might trigger. Cancer cells develop heterogenous resistance mechanisms to evade various drugs, but most of these mechanisms involve the reactivation of MEK–ERK signaling, combined with enhanced signaling output via the PI3K–AKT–mTOR and other pathways.^426^ Therefore, understanding the MAPK-related mechanisms underlying the development of resistance to RAFi and combination therapies may improve their potential therapeutic effects.^427,428^ Reactivation of ERK signaling can also occur via MAPK-independent pathways, including the bypassing of the paradoxical MAPK arm through PI3KC mutations,^429,430^ activation of an AKT–ETS-1–mediated positive feedback loop,^431^ loss of PTEN activity^432^ overexpression of mTOR,^433^ or manipulation of autophagy proteins (ATGs)^434,435^ (Fig. 8b, *MAPK-independent*). The combined inhibition of both the RAF–MEK and PI3K–AKT–mTOR pathways has a synergistic pro-apoptotic effect. For example, the addition of an AKT or mTOR inhibitor to Vemurafenib therapy or MEK inhibitor therapy results in synergistic effects.^436^ SHP2 inhibitors efficiently bypass the paradoxical MAPK signaling arm in *RAS*-mutant tumors treated with RAFi.^437,438^

#### The sequential or intermittent dosing of BRAF/MEK inhibitors

Patients with metastatic melanoma harboring activating BRAF mutations have demonstrated improved survival when receiving combination therapy with both BRAFi and MEKi.^439^ Unfortunately, the therapeutic effectiveness of such treatments often proves transient, with resistance emerging within a few months.^440^ Intermittent therapy has been explored as a strategy to halt or delay resistance to BRAF inhibition.^422^ In pursuit of curation approach for melanoma patients with BRAF mutations, researchers have proposed that modifying dosing patterns could prevent the development of lethal drug resistance and extend the durability of BRAFi responses.^441^ The optional implementation of this strategy in treating advanced BRAF^V600E^-mutant melanoma patients is currently under investigation in randomized phase II and III clinical trials comparing intermittent and continuous dosing schedules of BRAFi in combination with MEKi (*NCT02224781, NCT02196181*). However, one completed trial did not need its primary objective of improving progression-free survival, indicating that the experimental intermittent schedule of both Vemurafenib and Cobimetinib did not demonstrate superior anticancer activity compared to the regular continuous schedule (*NCT02583516*).

### Potential alternative strategies for further optimizing the use of RAF/MEKi

**Fig. 9:**
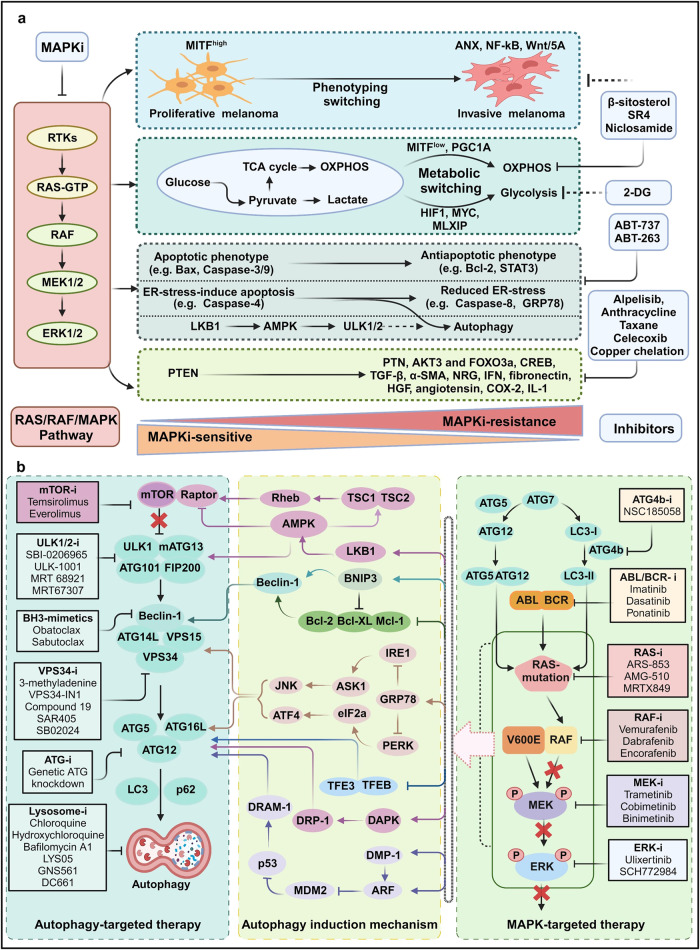
The possible therapeutic strategies for overcome resistance to RAS/RAF/MAPK inhibitors. **a** Alternative approaches to overcome MAPK inhibitor (RAFi) resistance. **b** Targeting autophagy. In cancer cells, autophagy-related genes are functionally and physically associated with mitogen-activated protein kinase (MAPK)-targeted therapy and cancer resistance induced by MAPK signaling inhibitors (e.g., RAFi, MEKi). The regulatory mechanisms involved in autophagy induction and the mediators that regulate autophagy are described in each rectangle. Arrows and bars indicate stimulating and inhibiting signals, respectively. This figure was created with BioRender.com

The potential of different combination therapies is being explored to delay the emergence of resistance to MAPKi or to precisely target BRAF/MEKi-resistance. Recent preclinical research has identified alternative strategies for combating resistance, including bolstering apoptosis, modulating autophagy, and directing attention on mitochondrial metabolism and phenotyping switch (Fig. 9a).

#### Targeting mitochondrial and energy metabolism

Metabolic disorders have long been associated with cancers, characterized by metabolic reprogramming, which involves alterations in metabolic pathways enabling cancer cells to proliferate rapidly, survive in hypoxia and nutrient-deprived conditions, and evade the immune system.^442^ Furthermore, metabolic reprogramming events play a crucial role in tumorigenesis, drug resistance, and metastases in melanomas.^443^ Research on chemotherapy-resistant, slow-cycling BRAF^V600E^ melanomas has shown increased level of oxidative phosphorylation (OXPHOS) enzymes and blocking them resulted in cell death. Additionally, the pivotal role of the microphthalmia-associated transcription factor (MITF) and PGC1A in regulating phenotyping plasticity is indispensable for steering cellular metabolism towards OXPHOS. A two-tiered strategy involves combining anticancer drugs that target rapidly proliferating melanoma cells with drugs that suppress the slow-cycling, drug-resistant subpopulation. Several drugs targeting mitochondrial respiration have been investigated in preclinical settings with promising results.^444^ For instances, β-sitosterol, due to its favorable tolerability, excellent bioavailability, and ability to inhibit mitochondrial respiration, serves as a viable adjuvant to BRAFi therapy for individuals with or at risk for melanoma brain metastases. Combining Vemurafenib with either β-sitosterol or a functional knockdown of mitochondrial complex I completely eliminated BRAFi resistance.^444^ Also, uncouplers like SR4 and Niclosamide that disrupt mitochondrial OXPHOS may be effective as first-line adjuvant treatments for melanoma patients who are not responding to MAPK inhibitors.^445^

Cancer starvation therapy, which relies on glucose restriction to induce oxidative stress and slow tumor growth has proven effective in curbing the rapid multiplication of cancer cells.^446^ The glucose analog 2-Deoxy-D-glucose (2-DG), when used in combination with Cisplatin or Erlotinib, enhances the cytotoxicity of head and neck squamous cell carcinoma (HNSCC) cells through metabolic oxidative stress. While 2-DG treatment alone may not cause significant cell death in most cancer cells, it does sensitize them to oxidative stress induced by radiotherapy or chemotherapy. Using 2-DG as an alternative therapeutic approach for the treatment of radioresistant and highly glycolytic cervical malignancies involves inhibiting intracellular redox metabolism and glycolysis.^447^ Also, some transcriptional factors, including hypoxia-inducible factor-1 (HIF1), MYC, and MONDOA (MLXIP), involve the regulation of glycolysis by inhibiting BRAF in melanomas, and further the combined inhibition of BRAF and glycolysis triggers cell death in BRAFi-resistant melanoma cells.^448^ Although there is currently no clinical evidence of this, targeting glucose metabolism could potentially be adapted therapeutically to overcome BRAFi resistance, particularly in pancreatic cancer driven by KRAS-dependent resistance to MAPK inhibition.^449^

Therapeutic modulation of metabolic difference in lipid metabolism can be a promising strategy to overcome BRAFi resistance. AMP-activated protein kinase (AMPK), a nutritional sensor present in both healthy and malignant cells, regulates lipid breakdown, phosphorylated, and inactivates acetyl-CoA carboxylase, preventing fatty acid production, and promotes lipid utilization through beta-oxidation in mitochondria.^450^ AMPK-dependent phosphorylation of BRAF at serine 729 reduces MAPK signal in *BRAF* wild-type cells by Inhibiting BRAF interaction with CRAF and KSR1.^451^ A study elucidated how 14-3-3 controls dimerization-driven RAF activation and mitigate the negative side effects of RAF inhibitors in cancer therapy.^452^ Additionally, the combination of Phenformin and PLX4720 resulted in tumor regression in some mice models. This study suggests that combining AMPK activators, such as Phenformin, with BRAF inhibitors may offer significant therapeutic advantages in melanoma treatment.

#### Targeting phenotyping switching

Phenotypic switching and metabolic reprogramming play distinct roles in the development of resistance in therapy-resistant clones of BRAF^V600^-mutated melanoma cells.^453^ The significance of MITF, a primary transcription factor in melanocyte differentiation/dedifferentiation, has been emphasized in relation to phenotypic switching. It has been observed that drug-sensitive cell lines and patient biopsies typically exhibit high MITF levels, whereas inherently resistant cell lines and patient biopsies demonstrated low MITF expression but elevated levels of NF-kB signaling and the receptor tyrosine kinase AXL.^454^ These MITF-low/NF-kB-high melanomas display resistance to ERK, RAF, and MEK inhibition in vitro. Remarkably, MITF expression show an inverse correlation with AXL expression, suggesting that cell lines can be categorized based on their metastatic potential regardless of whether they carry the BRAF or NRAS mutations.^455^ Additionally, Wnt5a signaling directly influences the motility and invasion of metastatic melanoma cells with low MITF levels.^456^ The phenomenon of EMT observed in cancer cells bears a striking resemblance to phenotypic switching.^457^ Exploring phenotypic switching may be essential in developing novel therapeutics capable of restoring sensitivity to RAFi or MEKi, providing a critical avenue for overcoming resistance.

#### Targeting the PI3K/AKT, tumor microenvironment and inflammatory responses

Resistance commonly develops in the majority of advancing melanomas through the reactivation of MAPK signaling, often driven by alterations in BRAF, NRAS, and MEK.^458^ While the activation of the PI3K/AKT pathway does not limit patient responses to BRAF/MEK inhibition, a small subset of resistant melanoma relies on the compensatory PI3K/AKT signaling cascade.^459^ Initial clinical trials with PI3K inhibitors in combination with Vemurafenib have shown promising results.^460^ In challenging-to-treat *BRAF*-mutant mCRC patients, a combination therapy comprising Alpelisib (a PI3K inhibitor), Encorafenib, and Cetuximab (an anti-EGFR) has demonstrated effectiveness.^461^ Notably, in *BRAF*^V600E^-mutated CRC cancers, activation of the PI3K/AKT pathway serves as a mechanism for both innate and acquired resistance to BRAF inhibitors, with proposed combination approaches to improve outcomes in this challenging patient population.^462^ PTEN, a major antagonist PI3K, is frequently mutated in various cancer tissues and is implicated in 17% of melanoma cases, 10% of colorectal cancer cases, and 4% of lung adenocarcinoma cases.^463^ Mutations in PTEN, resulting loss of function, are associated with a higher proportion of BRAF-mutant alleles, and are linked to lower progression-free survival (PFS), overall survival (OS), and response rates in melanoma patients. PTEN loss induces BRAFi-resistance in melanoma cells by inhibiting BIM expression.^355^ The involvement of AKT3 and the activation of FOXO3a play a role in enhancing apoptosis when PLX4720 and a PI3K inhibitor are combined to treat PTEN-negative cells.^355^ Importantly, HSP90 inhibition appears to be more effective in restoring BIM expression and downregulating Mcl-1 expression compared to the combined MEK/PI3K inhibitor therapy, making it a potentially highly effective strategy for managing the diverse array of resistance mechanism observed in BRAF resistance.^464^ AEBP1 (adipocyte enhancer-binding protein 1) is significantly upregulated in melanoma cells resistant to PLX4032 due to over-activation of the PI3K/AKT/CREB signal pathway. Blockage of this signaling pathway effectively restores the PLX4032-resistant phenotype of melanoma cells.

The development of resistance to BRAFi resistance in melanoma is a well-recognized challenge, stemming not only from genomic or epigenetic aberrations but also from the crucial role played by the tumor microenvironment. In response to BRAF inhibition, melanoma cells and fibroblasts undergo microenvironmental changes that enhance PI3K/AKT survival signaling, allowing tumor cells to evade treatment.^465^ When treated with Emurafenib, melanoma cells release TGF-β, triggering fibroblasts to increase expression levels of α-smooth muscle actin (α-SMA), neuregulin (NRG), and fibronectin. Paradoxically, Vemurafenib’s off-target effects lead to the secretion of hepatocyte growth factor (HGF) by fibroblasts. Furthermore, tumor-associated macrophages (TAMs) contribute to the development of a pro-tumorigenic microenvironment that fosters resistance to MAPKi therapy by providing an abundance of oxygen, nutrients (resulting in hypoxia and metabolic stress), and extracellular matrix proteins.^466^ Subsequently, TAMs secrete angiotensin, COX-2, IFN, and IL-1, further promoting the growth and metastasis of melanoma.^467^ Thus, to enhance the effectiveness of combination therapy for melanoma patients, targeting spatiotemporal interactions within tumor microenvironments holds significant promise.

Inflammatory signals establish a novel epigenetic program that silences a specific set of genes contributing to inflammation-induced cellular transformation in tumor cells.^468^ Tumor-induced inflammatory responses have adverse effects on the adaptive immune system and open up possibilities for new therapeutic strategies address the immunological dysfunctions caused by tumors.^469^ A novel independent predictor of Anthracycline/Taxane neoadjuvant chemotherapy response in breast cancer is the presence of tumor-associated lymphocytes, aiding healthcare professionals in identifying patients who will benefit most from this treatment.^470^ Pharmacological targeting of key factors derived from tumor-associated inflammation presents a unique strategy to eliminate therapy-resistant tumors. Celecoxib, a COX-2 inhibitor, significantly reduced tumor burden by 90% and notably delay tumor growth induced by the BRAFi inhibitor PLX7420.^471^ Copper chelation emerges as a potential treatment strategy for a specific subset of tumors characterized by activating BRAF^V600E^ mutations. Copper chelators, commonly used to treat Wilson’s disease, inhibit tumor formation in human or mouse cells harboring BRAF^V600E^ mutations or engineered to be resistant to BRAFi.^472^

#### Targeting apoptosis

Tumor cells often develop resistance to chemotherapy and radiation by disrupting the regulation of apoptosis-related mediators, especially by increasing the levels of anti-apoptotic BCL-2 family proteins.^473^ BH3 mimics have gained popularity as a means to induce cell death effectively by targeting the primary anti-apoptotic proteins without necessitating significant involvement of pro-apoptotic proteins.^474^ ABT-263, a BH3 mimic, when used in combination with selective BRAFi (PLX4720), enhances both the extent and speed of responses in treatment-naïve patients with *BRAF*^V600E^-mutated melanoma but it is less effective in individuals who have developed resistance to these drugs.^475^ Surprisingly, ABT-737 significantly increases the sensitivity of melanoma cell lines to conventional chemotherapeutics, leading to BIM-mediated apoptosis.^476^ Inhibiting anti-apoptotic BCL-2 proteins can improve primary PLX-4032 responses and reduces the development of resistance to both targeted and standard therapies.^477^

In addition to BCL-2 family proteins, STAT3 is a promising target for anti-apoptotic drug development against BRAFi resistance. Inhibiting the STAT3 pathway demonstrates significantly higher cytotoxicity compared to the currently used therapeutic drug PLX-4032. This approach targets both *BRAF*-mutant and WT melanoma cells without selectivity.^478^ Combing Vemurafenib with STAT3 silencing or miR-579-3p overexpression proves effective in overcoming Vemurafenib resistance in cancer cells.^479^ Furthermore, activation of the RAS-RAF-MAPK pathway is associated with the prevention of Caspase-3 activation, cell protection against apoptosis, and direct phosphorylation of caspase-9.^213^ Caspase-3 inhibits MEK1 through proteolytic means, leading to reduced pro-survival ERK signaling and increased susceptibility of cell to apoptosis.^480^

Endoplasmic reticulum (ER) stress can potentially activate various apoptotic signaling pathways in melanoma cells in a context-dependent manner. The MEK/ERK signaling pathway plays a pivotal role in preventing caspase-4 activation induced by ER stress. Additionally, the apoptosis repressor with caspase recruitment domain (ARC) proteins appears essential in preventing Caspase-8 activation in melanoma cells under ER stress.^481^ Inhibiting the MEK/ERK pathway makes melanoma cells more susceptible to apoptosis induced by ER stress, partially through caspase-4 activation, and is also associated with the inhibition of ER chaperon glucose-regulated protein 78 (GRP78) production.^482^

### Expanding current strategies using high-throughput technology

High-throughput techniques for profiling tumor-associated gene expression, including miRNAs, have a great prospect for clinical applications. A recent advancement involves a high-throughput quantitative method based on RCA, enhancing the sensitivity of detecting miRNAs associated with Triple-negative breast cancer (TNBC) using fluorescence-encoded microspheres.^483^

#### Genome editing

The translation of gene editing from theory to clinical practice has been accelerated by recent advancements in programmable nucleases, including zinc-finger nucleases (ZFNs), transcription activator-like effector nucleases (TALENs), and CRISPR/Cas9-associated nucleases.^484^ One of the pioneering pooled CRISPR screens involved a genome-scale knockout library of melanoma cells treated with the BRAF inhibitor, Vemurafenib, revealing genes responsible for treatment resistance.^485^ Another study found that CRISPR/Cas9 editing of RAS and MEK mutant cells led to resistance to BRAF and MEK inhibitors, but MEK1 Q56P restored sensitivity to the MEK/BRAF inhibitor combination, and KRAS G13D increased sensitivity to immunotherapy.^486^

#### Cancer vaccines

Cancer vaccines aim to activate latent or unresponsive tumor-specific T cells, bolstering the innate immune defense against cancer.^487^ Various cancer immunotherapies have been developed to trigger tumor-specific immune response in tumor patients. Clinical trials have demonstrated the effectiveness of cytosine-phosphorothioate-guanine oligodeoxynucleotides (CpG ODN) as a cancer vaccine adjuvant. The GO-PEI-OVA-PEG-CpG nano-vaccine exhibited favorable safety profiles and significant anticancer efficacy, extending mouse survival, and limiting tumor growth in vivo. When combined with NLG918 (antitumor immunotherapy), the vaccine displayed even greater therapeutic.^488^ Peptide-based drug delivery system, like cancer vaccines, offer an additional promising avenue to enhance existing BRAFi and MEKi strategies. An early-phase I clinical trial is therapy with the administration of six melanoma helper peptides (6MHP). The trial also assesses the impact of peptide vaccination, BRAF inhibitors, and MEK inhibitors on the immune system using participant blood and tumor samples. Initial results indicate that 77% of patients exhibited antibody responses to 6MHP by week 7, peaking 6 weeks after the last vaccine and persisting for 6 months (*NCT02382549*).

#### Leveraging online resources for compound screening

Resources for studying the RAF-centered signaling network are expanding, facilitating the discovery of protein sets that physically interact with MAPK network through high-throughput techniques, such as protein-interaction based screening approaches.^489^ The development of the NanoBRET screening platform employs live-cell bioluminescence resonance energy transfer (BRET) to identify substances that modify the binding between activated KRAS and CRAF kinase.^489^ Additionally, a subtractive forward two-hybrid method is employed to identify small molecule that disrupt the interaction between RAS and RAF.^490^ Furthermore, numerous online databases, including Cell Circuits, NCBI GEO, DIP, BIND, KEGG, NetPath, LINCS, BioGrid, and expert systems, enable individual researcher to construct and query protein interaction maps tailored to their gene of interest.

### Targeting autophagy: a new therapeutic avenue for RAS/RAF/MAPK inhibitor-resistance

Autophagy is generally affected by a wide range of anticancer medications, including DNA-damaging agents, microtubule-targeted therapies, antimetabolites, death receptor agonists, hormonal agents, antiangiogenic agents, proteasome inhibitors, histone deacetylase inhibitors, and kinase inhibitors.^262^ Both chemotherapeutic agents and ionizing radiation stimulate autophagy by inducing autophagosome formation.^262,491,492^ Some agents promote autophagosome formation but prevent lysosome fusion, consequently blocking autophagic flux.^493^ For example, mTOR inhibitors, such as Rapamycin and Temsirolimus, are well-known anticancer drugs that directly promote autophagy by disrupting the activity of the negative autophagy regulator mTOR complex 1 (mTORC1).^494^ ABT-737, a Bcl-2 inhibitor, directly targets the core autophagy machinery.^495,496^

Some autophagy inhibitors can attenuate tumor growth. Autophagy inhibition increases Icariin-induced cytotoxicity in colorectal cancer cells.^497^ In addition, several PI3K inhibitors, including 3-Methyladenine, Wortmannin, and LY294002, used as anticancer therapeutics, directly inhibit autophagy.^498,499^ Anticancer therapies that activate autophagy frequently induce a pro-survival response that may contribute to the development of drug resistance and refractory cancer.^500–503^ Therefore, drugs that target autophagy should be considered for combination therapy strategies together with other anticancer drugs. The antimalarial drugs Chloroquine (CQ) and Hydroxychloroquine (HCQ) are currently being studied as potential components of combination therapies together with standard treatments in multiple tumor types.^504–506^

#### Autophagy-associated RAFi resistance

Two recent studies have delved into the potential therapeutic benefits of combining autophagy inhibitors with targeted therapy.^507,508^ Based on a wealth of preclinical research, clinical trials, and recent literature, we have compiled a summary of autophagy-related RAFi-resistance and potential therapeutic interventions. MAPK inhibitors are used as conventional therapy in cancer patients with activated *RAF* mutations. However, their antitumorigenic effects and clinical benefits are temporary due to the eventual development of drug resistance and relapse. Drug resistance can occur when upstream factors activate these signaling pathways, bypassing RAF inhibition (Fig. 9b). The MAPK pathway is often aberrantly stimulated by BCR-ABL oncoproteins through direct binding with RAS activators, and aberrant MAPK activation plays significant roles in the onset and progression of leukemia.^509^ In addition, MAPK phosphorylation is frequently associated with autophagic structures, suggesting that microtubule-associated protein 1 A/1B-light chain 3 (LC3)-II–positive membranes and ATG5- and ATG12-positive pre-autophagosomes may serve as scaffolds or cellular signaling platforms for the RAF–MEK–ERK cascade, facilitating ERK pathway activation^510^. The ATG7–ATG5–ATG12–LC3-II cascade modulates MAPK phosphorylation in an unconventional manner (Fig. 9b). Atg4b, an LC3-specific protease, catalyzes the conversion of the LC3 precursor into its active forms and deconjugates modifiers from phospholipids.^511,512^

AMPK plays a crucial in promoting resistance to RAS-RAF-MAPK pathway inhibitors triggering autophagy.^513^ The oncogenic BRAF negatively regulates the tumor suppressor LKB1, fostering the proliferation of melanoma cells. Consequently, the RAS/RAF/MAPK pathway initiates autophagy activation through the LKB1-AMPK-ULK1 signaling axis. To induce mTORC1 inhibition and cell cycle arrest in response to energy stress, AMPK must phosphorylate Raptor, a part of mTORC1.^514^ The oncogenic BRAF leads to heightened basal autophagy and increased resistance to apoptosis in cutaneous melanomas, also causing chronic ER stress.^515^ ER stress-induced autophagy is diminished in cells lacking IRE1 or treated with a JNK inhibitor, highlighting the essential role of the IRE1-JNK pathway in autophagy activation following ER stress.^516^ Activation of the IRE1/ASK1/JNK and TRB3 pathways is induced by p38 activation, which is driven by BRAF^V600E^.^515^

BCL-2 family members (BCL-2, BCL-xL, and MCL-1) inhibit autophagy, whereas BNIP3 stimulates autophagy by releasing Beclin1 from the BCL2/Beclin1 complex.^496^ p53 family isoforms contribute to acquired resistance to targeted MAPK inhibitors in melanoma cells,^517^ and p53-dependent activation of damage-regulated autophagy modulator-1 (DRAM-1) triggers autophagy.^518^ *RAF*-mutant–induced chronic ER stress stimulates basal autophagy, and RAF-mediated p38 activation augments the IRE1–ASK1–JNK and Tribbles 3 (TRB3) pathways.^519^ DMP1 also acts as a critical molecule connecting oncogenic RAS/RAF/MEK/ERK signaling with the tumor-suppressive ARF–MDM2–p53 pathway.^520^ In conclusion, RAFi resistance in cancer is often associated with increased autophagy, driven by multiple interconnected pathways and molecular events, suggesting that understanding these mechanism may have implications for developing more effective treatment strategies in cancer.

#### Targeting autophagy to overcome RAFi resistance as a therapeutic strategy

BRAF^V600E^-mutant MAPK activation in cancer highlights the potential therapeutic benefits of using MAPK inhibitors, such as Vemurafenib, which have demonstrated beneficial outcomes when used to treat patients with late-stage melanoma.^324^ In addition, pharmacological inhibitors of *BRAF*^*V600E*^-mutant and MEK have been used to treat metastatic carcinoma.^331,337,521^ However, the rapid development of drug resistance has limited the use of these drugs in cancer therapy, resulting in temporary benefits lasting from several months to less than 2 years.^522^ When combined with immune checkpoint inhibitors targeting programmed death protein 1 or ipilimumab targeting cytotoxic T lymphocyte–associated protein 4, these medications greatly enhance life expectancy up to 4 years in 50% of patients with metastatic melanoma.^523,524^ However, combination therapy using RAFis or MEK inhibitors together with immune checkpoint inhibitors remains restricted to patients with certain types of cancer.^525–528^ To enhance therapeutic efficacy, a wide range of adaptive responses must be studied, including therapy resistance mechanisms and autophagy, which enable drug sequestration and cell survival.

##### Inhibition of adaptive protective autophagy induced by MAPK re-sensitization in RAFi resistance

Numerous avenues that lead to acquired resistance to RAFis generally utilize multiple methods to target the same or parallel pathways.^366^ Vertical inhibition of the MAPK signaling pathway using combinations of RAFi together with MEK inhibitors has received FDA approval as a first-line treatment strategy for patients with advanced *RAF*-mutant melanoma, NSCLC, and thyroid cancer. Suppression of *RAF*-mutant signaling promotes autophagy in cancer cells,^25,26^ suggesting that targeting autophagy in cancer cells may be desirable when using pathway-targeted inhibitors in *RAF*-mutant cancer.^529^ Many tumors harboring *RAS* and *RAF* mutations develop an “addiction” to autophagy, which is required to maintain cellular homeostasis; thus, the inhibition of protective autophagy induced by MAPK pathway activation may represent a therapeutic option in *RAS-* and *RAF*-mutant malignancies.^435,530–533^ The genetic suppression of autophagy genes may also represent a novel approach for the treatment of lung cancers harboring mutant *RAS*^534^ or *BRAF*^*V600E*^.^535^ Pharmacologic RAF inhibition dramatically increases autophagy in RAFi-resistant melanoma cells,^26^ and the genetic or pharmacological inhibition of autophagy counters RAFi resistance in brain tumors.^434^ Additionally, inhibiting autophagy in RAFi-resistant tumor cells in vitro and in patients reverses drug resistance.^434^ Combining autophagy inhibition with MEK inhibition increases apoptotic cell death in a *RAF*-mutant colorectal cancer cell line.^536^ The combined inhibition of autophagy and MEK significantly accelerates regression in *RAF*-mutant patient-derived colorectal cancer cells xenografted onto an animal model.^537^ Similarly, *RAF*-mutant thyroid cancer cells are sensitive to autophagy targeting in the presence of vemurafenib.^320,538^

#### Therapeutics success beyond autophagy targeted RAFi therapy

In cancer, autophagy plays adaptive and protective roles when MAPK signaling is hampered.^539^ Accordingly, combined treatment using both MAPK and autophagy inhibitors represents one of the best options for treating various carcinomas.^26,531^ However, tumor responses to autophagy inhibitors are not clinically consistent due to non-uniform permeability across tumor tissues and the potential toxicity of combination treatments using multiple chemotherapeutics.^540^

Because autophagy can act as a double-edged sword during tumorigenesis, autophagy-targeted treatment outcomes are highly dependent on cancer types, contexts, and stages.^541,542^ In addition, the inhibition of autophagy initiation and late fusion steps results in differential responses. For example, early-stage autophagy inhibition using ULK1/2 inhibitors (e.g., MRT 68921) decreases tumor cell death, whereas inhibitors (HCQ) that target the later fusion stage greatly increase cell death in mesothelioma cells.^543^ CQ and its variants, such as HCQ, are currently the most frequently tested autophagy inhibitors in cancer clinical trials. These chemicals impede the late fusion stage between autophagosomes and lysosomes, causing deacidification and impairing enzymatic function.^540,544,545^ Interestingly, these inhibitors lack selectivity, affecting total lysosomal function.^546^ However, CQ largely improved the sensitivity of Vemurafenib in an ex vivo primary cell culture derived from patients harboring the *BRAF*^V600E^ mutation^435^. Autophagy-independent vascular normalization of CQ inhibits tumor invasion and metastasis by enhancing chemotherapeutic effects.^547,548^ The autophagy inhibitor HCQ has been more widely used in clinical trials over CQ because HCQ is less toxic at optimum doses.^504,549–551^ HCQ shows beneficial effects and synergistic effects when combined with the mTOR inhibitor, Temsirolimus, in melanoma^552^ and breast cancer.^553,554^ Importantly, HCQ affects chemotherapy and radiation sensitivity in non-selective cancers.^555^ Lys05, a novel lysosomal autophagy inhibitor, has also been suggested as a potential therapeutic agent for cancer.^556,557^

CQ and HCQ are non-selective autophagy inhibitors, and specific small-molecule inhibitors that target earlier stages of autophagy are preferred for cancer therapy.^547,558–560^ ULK1, a key autophagy regulator, can be specifically targeted by cellular energy status regulators, such as mTORC1 and AMPK.^561^ AMPK, a low-energy sensor, activates ULK1 to induce autophagy.^562^ ATG13, an autophagy protein in the ULK1 complex, is a static autophagy marker that corresponds closely with autophagic flux in mesothelioma.^563^ In addition, multiple ATP-competitive ULK1 kinase inhibitors have demonstrated compelling evidence to support clinical use,^564,565^ such as the successful suppression of NSCLC growth by SBI-0206965.^565,566^ Vacuolar protein sorting 34 (VPS34), a component of the Beclin1 complex, also plays an important early role in autophagosome formation and is considered a potential therapeutic target.^567,568^ Several VPS34 inhibitors (e.g., SAR405) have demonstrated anticancer effects in kidney carcinoma cells,^569^ including VPS34-IN1, a particularly powerful and selective VPS34 inhibitor with cancer therapeutic potential.^570^ Furthermore, transitional small molecules tend to form prolonged associations with target proteins, leading to the inhibition of their enzymatic activity. This extended binding duration poses challenges in devising small molecule structures and countering drug resistance resulting from target mutations. In contrast, the proteolysis-targeting chimera (PROTAC) exhibits the ability to specifically eliminate target proteins, thereby intensifying the cytotoxic impact on mutant tumors. In the realm of both fundamental research and pharmaceutical advancement, the autophagy-targeting chimera (AUTOTAC) provides a versatile platform for precise proteolysis.^571^ Also, high-throughput technology presents a promising approach for identifying selective autophagy inhibitors that are both effective and less cytotoxic when used in combination therapy. Arzonol, for instance, emerged as a distinct chemotherapeutic candidate through high-throughput screening of natural compounds aimed at modulating autophagy.^572^ Overall, targeting autophagy initiation, together with improved investigations of the functional mechanisms underlying existing chemotherapies, could be crucial for developing novel cancer therapies.

## Conclusion and future directions

RAF inhibitors exhibit exceptional clinical efficacy in patients with *RAF*-mutant carcinoma, although their therapeutic effects are restricted due to the development of drug resistance. Recent in vitro and in vivo translational studies have elucidated the molecular basis underlying the development of RAFi resistance in cancer. Obtaining additional biological insights into the process of drug resistance represents a necessary, long-term goal for the development of new RAFi and combination treatments able to slow or prevent the development of drug resistance in cancer. The combined regulation of MAPK signaling and RAFi-induced autophagy may represent a potential strategy for overcoming drug resistance and inform the development of novel cancer therapies. Because autophagy can act as either a pro-death or a pro-survival factor in tumorigenesis, targeting autophagy is highly dependent on cancer type and stage. Autophagy activation can promote tumorigenesis, and autophagy inhibition can improve the therapeutic efficacy of RAFi. A better understanding of the molecular mechanisms and precise targets underlying the complex process of autophagy modulation could lead to the development of agonists or blockers that could serve as potential therapeutic agents for future use in *RAF*-mutant cancers.

Pharmacological autophagy inhibitors hold significant potential in combating resistance to RAF inhibitors in cancer patients with RAF mutations. Recent research highlights the efficacy of compounds like 3-Methyladenine and hydroxychloroquine (HCQ) in suppressing autophagy when combined with anti-EGFR monoclonal antibodies (mAbs) and checkpoint inhibitors. The BAMM trial (BRAF Autophagy and MEK Inhibition in Melanoma), which combined Dabrafenib, Trametinib, and HCQ, demonstrated these combinations are effective and safe in patients with BRAF^V600^-mutant melanoma. Beyond classical autophagy inhibitors, emerging indirect inhibitors like ABT-737, natural products such as Resveratrol, and synthetic compounds like Quinacrine may warrant clinical investigation. The AUTOTAC chemical biology platform has garnered significant attention within the area of targeted protein degradation. It employs bifunctional molecules that consist of ligand binding to specific targets linked with ligands targeting the autophagy processes. Furthermore, recent advancements in autophagy-based degraders and molecular adhesives, such as autophagosome-tethering compounds (AATEC) or lysosome-targeting chimera (LYTAC), have enabled the successful degradation of numerous target proteins by routing them to the lysosome.

Also, the genetic inhibition of autophagy genes via RNA interference and CRISPR/Cas9 targeting holds promising. Evaluating the safety of combing BRAF and MEK inhibitor therapy with melanoma helper peptides (6MHP) administration assesses the impact on the immune system. In addition, cancer vaccines aim to activate tumor-specific T cells, strengthening the immune defenses. Future research emphasizes high-throughput technology for preclinical evaluations of cytotoxicity and combination therapy between RAS/RAF/MAPK inhibitors and other compounds.
